# Functional characterisation of three members of the *Vitis vinifera* L. carotenoid cleavage dioxygenase gene family

**DOI:** 10.1186/1471-2229-13-156

**Published:** 2013-10-09

**Authors:** Justin G Lashbrooke, Philip R Young, Samantha J Dockrall, Krishnan Vasanth, Melané A Vivier

**Affiliations:** 1 Institute for Wine Biotechnology, Department of Viticulture and Oenology, Stellenbosch University, Private Bag X1, Matieland, 7602, South Africa; 2 Current address: Department of Botany, Bharathiar University, Coimbatore, TN, 641 046, India

**Keywords:** *Vitis vinifera* L, Grapevine, Carotenoid, Apocarotenoid, Cleavage dioxygenase, CCD1, VvCCD4

## Abstract

**Background:**

In plants, carotenoids serve as the precursors to C_13_-norisoprenoids, a group of apocarotenoid compounds with diverse biological functions. Enzymatic cleavage of carotenoids catalysed by members of the carotenoid cleavage dioxygenase (CCD) family has been shown to produce a number of industrially important volatile flavour and aroma apocarotenoids including β-ionone, geranylacetone, pseudoionone, α-ionone and 3-hydroxy-β-ionone in a range of plant species. Apocarotenoids contribute to the floral and fruity attributes of many wine cultivars and are thereby, at least partly, responsible for the “varietal character”. Despite their importance in grapes and wine; carotenoid cleavage activity has only been described for VvCCD1 and the mechanism(s) and regulation of carotenoid catabolism remains largely unknown.

**Results:**

Three grapevine-derived CCD-encoding genes have been isolated and shown to be functional with unique substrate cleavage capacities. Our results demonstrate that the VvCCD4a and VvCCD4b catalyse the cleavage of both linear and cyclic carotenoid substrates. The expression of *VvCCD1*, *VvCCD4a* and *VvCCD4b* was detected in leaf, flower and throughout berry development. *VvCCD1* expression was constitutive, whereas *VvCCD4a* expression was predominant in leaves and *VvCCD4b* in berries. A transgenic population with a 12-fold range of *VvCCD1* expression exhibited a lack of correlation between *VvCCD1* expression and carotenoid substrates and/or apocarotenoid products in leaves, providing proof that the *in planta* function(s) of VvCCD1 in photosynthetically active tissue is distinct from the *in vitro* activities demonstrated. The isolation and functional characterisation of VvCCD4a and VvCCD4b identify two additional CCDs that are functional in grapevine.

**Conclusions:**

Taken together, our results indicate that the three CCDs are under various levels of control that include gene expression (spatial and temporal), substrate specificity and compartmentalisation that act individually and/or co-ordinately to maintain carotenoid and volatile apocarotenoid levels in plants. Altering the expression of *VvCCD1* in a transgenic grapevine population illustrated the divergence between the *in vitro* enzyme activity and the *in planta* activity of this enzyme, thereby contributing to the efforts to understand how enzymatic degradation of carotenoids involved in photosynthesis occurs. The identification and functional characterisation of VvCCD4a and VvCCD4b suggest that these enzymes are primarily responsible for catalysing the cleavage of plastidial carotenoids.

## Background

Carotenoids are hydrophobic C_40_ isoprenoid pigments occurring throughout the natural world. In higher plants carotenoids are typically associated with photosynthetic membranes of plastids, especially the chloroplasts where they are involved in a number of photochemical reactions. In chloroplasts carotenoids primarily act as accessory pigments in the light harvesting antennae complexes and also assist in photoprotection by quenching free radicals and preventing photo-oxidative damage to the cell. The carotenoids in chloroplasts are typically not found “free” within the aqueous stroma, but rather bound in distinct pigment-protein antenna complexes within the membranes of the thylakoids (grana) [1].

The localisation and composition of carotenoids change during stresses (e.g. the xanthophylls cycle), and during plastid transitions/differentiation: chloroplasts to chromoplasts (as occurs in some fruits and flowers) or chloroplasts to gerontoplasts (as occurs during senescence) [2]. The genes and enzymes involved in carotenoid biosynthesis in plants have received much attention and the conserved pathway is relatively well characterised (reviewed in [3,4]); similarly the genes and enzymes of the carotenoid metabolic pathway for *V. vinifera* have been described previously [5,6].

The carotenoid cleavage dioxgenase (CCD) enzyme family contribute to the directed enzymatic production of apocarotenoids. Apocarotenoids and their derived metabolites have diverse biological roles (reviewed in [7]). In *Arabidopsis thaliana* nine different CCDs have been identified [8]. Five of the *Arabidopsis* CCDs (NCED2, NCED3, NCED5, NCED6, and NCED9) catalyse the first step towards abscisic acid (ABA) biosynthesis [9]. The remaining four *Arabidopsis* members of this protein family (CCD1, CCD4, CCD7 and CCD8) have more divergent activities and catalyse the cleavage of a variety of carotenoid substrates at specific double bond positions. Sequential cleavage catalysed by CCD7 and CCD8 are involved in formation of the shoot-branching inhibiting hormone, strigolactone [10]. CCD1- and CCD4-catalysed cleavage results in the production of a variety of flavour and aroma compounds [11].

The CCD1 orthologues are highly similar and are the only members of the CCD enzyme family predicted to have a cytosolic (rather than plastidial) localisation [8]. Orthologues have been shown to catalyse the symmetrical cleavage of a variety of carotenoids *in vitro* at the 5,6 (5’,6’) and 9,10 (9’,10’) double bond position producing a wide range of volatile C_13_-norisoprenoids. These carotenoid-derived C_13_-norisoprenoids are found in flowers, fruits, and leaves of many plants [12,13] and are considered to be flavour and aroma compounds, often displaying very low detection thresholds. Due to their contribution to the fruity and floral varietal perception of wine; C_13_-norisoprenoids have received much interest in wine grapes ([14-16] and reviewed in [17]). Previous attempts to determine the role of CCD1 in plants through transgenic manipulation of its transcript levels have been performed in *Solanum lycopersicum* (in tomato fruit) [18]; *Medicago truncatula* (in mycorrhizal roots) [19]; and *Oryza sativa* (in rice endosperm) [20]. These studies suggested that the observed *in vitro* functioning of CCD1 (i.e. catalysis of the symmetrical 9,10 (9’,10’) oxidative cleavage of carotenoids) may not be its sole biological action *in planta* and that other genes may code for enzyme(s) which are primarily responsible for catalysing this cleavage reaction in plastids [21].

To date CCD4 orthologues have only been identified in Angiosperms (flowering plants) and characterisation of CCD4 enzyme activity has shown that these members of the CCD family contribute to the carotenoid-derived flavour and aroma profile of both fruits and flowers. Subcellular localisation studies in *A. thaliana*[22] and *Crocus sativus* (saffron) [23] have demonstrated that the enzymes are targeted specifically to the plastoglobules within the plastids. Unlike the cytosolic localisation of the CCD1 orthologues; the CCD4 localisation gives it access to its carotenoid substrates [24]. CCD4 enzymes often occur as two isoforms: CCD4a and CCD4b that share modest similarity to each other and have distinct expression patterns and potentially divergent functions in plants. Based on reports from various plants there seems to be no consensus on the specific enzyme activity of the CCD4 enzymes.

Although CCD1 and CCD4 enzymes catalyse the cleavage of carotenoids at the same double bond positions; CCD4 enzymes are proposed to be substrate-specific, whereas the CCD1 enzymes are commonly described and demonstrated as more promiscuous in their substrate preferences [25]. A study by Huang *et al.*[11] concluded that CCD4s cannot catalyse the cleavage of linear carotenoids (e.g. lycopene and *cis*-ξ-carotene), or carotenoids that contain hydroxyl groups (e.g. zeaxanthin and lutein) and the current hypothesis is therefore that CCD4 orthologues catalyse cyclic non-polar carotenoid cleavage (e.g. β-carotene).

By identifying, isolating and characterising the grapevine *CCD*s, putatively involved in flavour and aroma related apocarotenoid production, the current work aimed to elucidate the biological role of these genes and their respective enzymes in grapevine. This study reports the isolation of CCD1, CCD4a and CCD4b-encoding genes from *Vitis vinifera* L. cv Pinotage. The CCDs were characterised by determining their expression patterns and functionality in grapevine. Functionality was described by analysing the volatile apocarotenoids formed following carotenoid cleavage in a heterologous *Escherichia coli* (bacterial) system. Furthermore, a population of transgenic grapevine (*V. vinifera* L. cv. Sultana) with altered expression of *VvCCD1* (up-regulated and down-regulated) was generated and characterised genetically and phenotypically. Leaf tissue of the transgenic Sultana lines was analysed via HPLC and GC/MS for the detection and quantification of carotenoids and volatile apocarotenoids, respectively.

## Methods

### *In silico* analyses

The National Center for Biotechnology Information (NCBI) Entrez search and retrieval system was used to obtain nucleotide and protein sequences from the Genbank databases (http://www.ncbi.nlm.nih.gov/gquery). Alignments to sequences in the Genbank databases were performed using the relevant Blast algorithm (http://www.ncbi.nlm.nih.gov/BLAST/) [26]. Comparative genomics (i.e. gene structure prediction and homologue/orthologue retrieval) were performed via PLAZA (http://bioinformatics.psb.ugent.be/plaza/) [27].

The putative sub-cellular localisation of protein sequences were predicted using ProtComp Version 8.0 (http://www.softberry.com/berry.phtml). *V. vinifera* expressed sequence tags (ESTs) were retrieved from The Institute for Genomic Research (TIGR) Grape Gene Index (http://compbio.dfci.harvard.edu/tgi/) or NCBI. The *V. vinifera* genomic sequences were retrieved from NCBI or Genoscope (http://www.cns.fr/externe/GenomeBrowser/Vitis/). Protein alignments were performed with Clustal Omega (http://www.ebi.ac.uk/tools/msa/clustalo/), and the resultant phylogenetic trees visualised using MEGA [28].

#### Plant material

Grapevine material for gene isolation, native expression analysis and pigment analysis was harvested from field-grown *Vitis vinifera* L. cv. Pinotage at Welgevallen experimental farm (Stellenbosch, South Africa). Green, véraison and ripe berries, as well as fully expanded leaf and mature flower material were harvested and flash frozen in the field in liquid nitrogen. The frozen tissue was homogenised in liquid nitrogen and, if not used immediately, stored at -80°C.

Transgenic plants were housed in a greenhouse and grown in a commercial soil mixture supplemented with Nitrosol® every 3 weeks. Analyses of gene expression, pigment concentrations and volatile composition were performed on tissue from fully expanded leaf tissue (leaf position 3 and 4). Leaves were flash frozen in liquid nitrogen immediately upon harvesting and stored in the dark at -80°C. Control plants underwent the same tissue culture, hardening-off and glass house conditions and procedures as the transgenic population.

### Isolation, extraction and manipulations of nucleic acids

High molecular weight genomic DNA was isolated from fully expanded *V. vinifera* leaves as described by Steenkamp *et al.*[29]. Total RNA from different grapevine leaves was extracted according to the methods described by Reid *et al.*[30]. Unless otherwise stated, all standard methods for plasmid DNA isolation, manipulations and cloning of DNA fragments, and agarose gel electrophoresis were used as described by Sambrook *et al.*[31].

Total cDNA was synthesised from 1 μg DNase I-treated (Promega, Madison, WI) total RNA using the Superscript III Platinum first strand synthesis system (Invitrogen) in a 20 μL reaction volume as described by the supplier.

### Bacterial strains, media, growth conditions, and transformations

*Escherichia coli* (DH5α and TOP10F) and *Agrobacterium tumefaciens* (EHA105) cultures were grown in LB media (1.2% (w/v) tryptone, 1.2% (w/v) NaCl and 0.6% (w/v) yeast extract). Bacterial transformations were performed using the heat-shock method as described in Sambrook *et al.*[31]. Transformants were selected using the appropriate antibiotic as selection on LB plates. Putative positive colonies were cultured and their plasmids isolated and verified by restriction digest. Unless otherwise stated all *E. coli* cultures were grown at 37°C and *A. tumefaciens* cultures at 30°C.

### Plasmids, cloning and bacterial transformations

The carotenoid accumulating strains used to perform the functional complementation assays were obtained from F. X. Cunningham (Department of Cell Biology and Molecular Genetics, University of Maryland, MD, USA) and are described in Cunningham [4] and Cunningham *et al.*[32,33].

The primer pair VvCCD1_5’ and VvCCD1_3’ was used to amplify the *VvCCD1* gene from *V. vinifera* L. cv. Pinotage cDNA (Additional file 1). The primer pairs VvCCD4a_5’, VvCCD4a_3’ and VvCCD4b_5’, VvCCD4b_3’ were used to amplify *VvCCD4a* and *VvCCD4b* from *V. vinifera* L. cv. Pinotage cDNA (Additional file 1). The resultant PCR amplicons were cloned into the pGEM-T Easy vector system according to the specifications of the supplier (Promega), to generate the plasmids pGEMt-VvCCD1, pGEMt-VvCCD4a and pGEMt-VvCCD4b (Additional file 2).

For the construction of plasmids for bacterial functional complementation, the 1715 bp VvCCD1 coding region was excised from pGEMt-VvCCD1 as an *Nde*l/*Pst*I fragment and cloned into the corresponding sites of pTWIN1 to yield pTWIN1-VvCCD1. The 1722 bp VvCCD4a coding region was excised from pGEMt-VvCCD4a as *Nde*l/*Bgl*II fragment and cloned into the compatible *Nde*l*/Bam*HI sites of pTWIN1 to yield pTWIN1-VvCCD4a. The 1770 bp VvCCD4b coding region was excised from pGEMt-VvCCD4b as an *Nde*l*/Bam*HI fragment and cloned into the corresponding sites of pTWIN1 to yield pTWIN1-VvCCD4b (Additional file 2).

The binary vector, pART27 was used for both the overexpression and silencing constructs [34]. The 1715 bp VvCCD1 coding region was excised from pGEMt-VvCCD1 as a *Sal*I/*Spe*I fragment and cloned into the compatible *Xho*I/*Xba*I sites of pART7 to yield pART7-VvCCD1. The expression cassette was excised with *Not*I and cloned into the corresponding site of pART27 to yield pART27-VvCCD1.

The pHANNIBAL vector was used for the construction of a VvCCD1 RNAi/silencing vector [35]. A 148 bp fragment was PCR-amplified from the 3’ untranslated region (UTR) of *VvCCD1* from *V. vinifera* L. cv Pinotage genomic DNA using the primer pair VvCCD1_RNAi_5’ and VvCCD1_RNAi_3’ (Additional file 1). The pGEM®-T Easy vector system was used to clone the PCR amplicon according to the specifications of the supplier (Promega), creating the pGEMt-CCD1(RNAi) plasmid. A 136 bp *Xho*I and *Eco*RI fragment was excised from pGEMt-CCD1(RNAi) and ligated into the corresponding *Xho*I and *Eco*RI sites in pHANNIBAL. The resultant plasmid was subsequently digested with *Bam*HI and *Xba*I and the 148 bp *Bam*HI and *Xba*I fragment from pGEMt-CCD1(RNAi) was ligated into the corresponding sites. The resultant plasmid, pHANNIBAL-CCD1(RNAi), contained a 148 bp inverted repeat of the 3’-UTR of *VvCCD1*. The expression cassette was excised from pHANNIBAL-CCD1(RNAi) with *Not*I, and ligated into the corresponding *Not*I site of pART27 yielding the final *VvCCD1* silencing vector, pART27-CCD1(RNAi) (Additional file 2).

### Grapevine transformation and regeneration

Somatic embryogenic cultures of *V. vinifera* L. cv. Sultana were used as source material for the genetic transformation experiments. The somatic embryogenic cultures were obtained and maintained according to the methods described in Vasanth and Vivier [36]. The genetic transformation protocol was essentially according to Franks *et al.*[37] with some modifications to use liquid cultures as starting material (as described in Vasanth and Vivier [36]). Briefly, *Agrobacterium tumefaciens* cells, containing either the overexpression or silencing vector, were harvested by centrifugation at 5,000 rpm for 10 min and resuspended in liquid NN medium, containing 18.5 μg.mL^-1^ maltose to a final OD_600_ of 0.8. Acetosyringone (19.7 mg.L^-1^) was added to the agrobacterial suspension before 2 mL of the somatic embryogenic cell suspensions were added and left for 15 min, with gentle shaking three to five times during this period. The culture was filtered to remove the excess liquid and the callus blotted dry using sterile Whatman no.1 filter paper. Co-cultivation proceeded at 27°C in the dark for 2 days on solid NN medium supplemented with BAP (0.25 μg.mL^-1^), NOA (1.0 μg.mL^-1^) and acetosyringone (19.7 μg.mL^-1^). Subsequently embryos were washed with sterile NN medium containing carbenicillin (200 μg.mL^-1^), blotted dry on sterilised filter paper and handled according to the protocol of Franks *et al*. [37]. Selection on kanamycin (100 μg.mL^-1^) was maintained until *in vitro* rooted plantlets were obtained, and subsequently hardened off in a greenhouse.

### Southern blot analysis

Southern blot analysis was performed using 10–20 μg of genomic DNA extracted from grapevine leaves. The DNA was digested with *Spe*I, separated in a 0.8% (w/v) TBE agarose gel and transferred to a positively charged Hybond-N nylon membrane as described by the supplier (Amersham-Pharmacia Biotech, Buckinghamshire, UK). Probe labelling, hybridisation, and Biotin detection were performed using the Biotin non-radioactive nucleic acid labelling and detection system according to the specifications of the supplier (Roche Diagnostics, Mannheim, Germany).

### Expression analysis of *VvCCDs*

Primers for qRT-PCR for the expression analysis of *VvCCD1, VvCCD4a, VvCCD4b, VvCCD4c, VvCCD4d, VvCCD7* and *VvCCD8* were designed using Primer Express 3.0 (Applied Biosystems) (Additional file 1). The *V. vinifera* elongation factor 1α (*VvEF1α*) was selected as a “house-keeping” gene to normalise gene expression based on the findings of Reid *et al.*[30] and Guillaumie *et al.*[38]; Relative expression analysis of the *VvCCD* gene family was performed in three different berry developmental stages, corresponding to green, vérasion and ripe berry stages of *V. vinifera* L. cv Pinotage.

Expression analysis of *VvCCD1* in the transgenic grapevine population was similarly performed via qRT-PCR. The grapevine glyceraldehyde-3-phosphate dehydrogenase (*VvGAPDH*) gene was used as a “house-keeping” gene to normalise gene expression. The expression of *VvGAPDH* has been shown to be relatively invariant in grapevine berries [30]. Primers were designed to evaluate total *VvCCD1* expression (i.e. endogenous and transgene-derived expression), as well as expression derived from only the transgenic *VvCCD1* (Additional file 1).

Real-time PCR was performed using an Applied Biosystems 7500 Real-time PCR System. KAPA SYBR® FAST qRT-PCR Kit was used according to the manufacturer’s (Kapa Biosystems, Cape Town, South Africa) instructions. The programme for the PCR reactions was: 50°C for 2 minutes; 95°C for 10 minutes; and 40 cycles of 15 seconds at 95°C and 60 seconds at 58°C. Data were analysed using the Applied Biosystems SDS software (version 1.4). All PCR reactions consisted of at least three technical replicates. Relative expression was calculated using the equation as described by Pfaffl [39]:

$${\left(E_{\mathrm{target}}\right)}^{\Delta \mathrm{CPtarget}\;\left(\mathrm{control}-\mathrm{sample}\right)}/{\left(E_{\mathrm{reference}}\right)}^{\Delta \mathrm{CPreference}\;\left(\mathrm{control}-\mathrm{sample}\right)}$$

(where *E* is the PCR efficiency and CP is the cycle number that the florescence crosses the base line).

### Bacterial functional complementation and determination of volatile apocarotenoids in bacterial headspace

The extraction and analysis of the volatile apocarotenoids from bacterial cultures were performed based on a method described in Lücker *et al.*[40]. For clarity the method and relevant modifications are described in detail. pTWIN1-VvCCD1, pTWIN1-VvCCD4a and pTWIN1-VvCCD4b plasmids were introduced into carotenoid accumulating *E. coli* strains. An empty vector, pTWIN1, was used as a negative control. An overnight culture (5 mL) grown in LB media to saturation was used to inoculate 32 mL of LB containing the appropriate antibiotics (100 μg.mL^-1^ ampicillin, 34 μg.mL^-1^ chloroamphenicol and 12.5 μg.mL^-1^ tetracycline) until an OD_600nm_ of 0.1 was reached. The cultures were incubated in the dark, gently shaking at room temperature until an OD_600 nm_ of 0.6 was reached. To prevent further production of coloured carotenoids the inhibitor diphenylamine (DPA) was added to a final concentration of 100 μM (as described in Cunningham and Gantt [41]) and the cultures were incubated at room temperature for an additional two hours in the dark. After the two hour incubation with the inhibitor, 8 mL of the 32 mL culture was removed and flash frozen for carotenoid analysis; and the remaining 24 mL of culture was harvested for apocarotenoid analysis. For apocarotenoid analysis the cells were resuspended in 6 mL of LB containing the appropriate antibiotics, 0.1 mM isopropyl-β-D-thiogalactopyranoside (IPTG), and 1 ppm of α-terpineol (as internal standard, IS). In addition 6 mM ascorbate, 5 μM ferrous sulphate, 200 U/mL catalase were added as described in Baldermann *et al.*[42] for *in vitro* CCD1 enzyme assays. The cultures were subsequently transferred to 20 ml SPME vials. The vials were sealed with Bi-metal®crimp seals with 20 mm silicone/polytetrafluoroethylene (PTFE) septa (Brown Chromatographic supplies, Wertheim, Germany). The samples were incubated in the dark, gently shaking at room temperature for 16 hours. After 16 hours, 1 mL of culture was removed for OD_600nm_ determination and 5 mL of 5 M NaCl was added to the 5 mL remaining culture for volatile apocarotenoid extraction.

The HS-SPME extraction of volatile apocarotenoids from the bacterial cultures was performed using a CTC CombiPal auto sampler equipped with the SPME option (CTC Analytics, Switzerland). Extraction conditions were as follows: after incubation at 50°C for 2 minutes SPME extraction was performed for 15 min under constant agitation by exposing a divinylbenzene/Carboxen/polydimethylsiloxane (DVB/CAR/PDMS) SPME fibre (Supelco, Bellefonte, PA) to the headspace. Thereafter the fibre was desorbed and analytes subsequently injected onto the GC column using a split/splitless injector, operated at 240°C, splitless for 2 minutes. The fibre was left in the injector for a further 20 minutes at 270°C for conditioning of the fibre under a purge flow of 60 mL/min. Separation of compounds was achieved on a DB-FFAP column (60 m × 0.25 mm × 0.5 μm) on an Agilent 6890 gas chromatograph coupled to an Agilent 5975C mass spectrometer (MS) (Agilent Technologies, Little Falls, Wilmington, USA). The helium carrier gas flow through the column was 1.2 mL/min and the oven programmed from 40°C (held 5 min), ramped at 10°C/min to 230°C (held for 2 min), with a post run at 240°C (held for 2 min). The total run time was 30 minutes.

The MS was operated in electron impact (EI) mode (70 eV) using Selected Ion Monitoring (SIM), simultaneously acquiring scan data as well. In SIM mode the following m/z fragments were monitored: α-terpineol (IS) (59, 93, 136), 6-methyl-5-hepten-2-one (MHO) (69, 111, 126), geranyl acetone (69, 136, 121), α-ionone (121, 136, 192), β-ionone (136, 177, 192) and pseudo-ionone (81, 109, 135). Compound identification was performed by both comparisons of the retention times with that of authentic standards and the NIST2005 mass spectral library (National Institute of Standards, USA). Peaks of interest were quantified by external standard curves of the authentic standards. Data were normalised to the internal standard concentration (α-terpineol) and to the OD_600_ of the bacterial cultures before HS-SPME analysis.

### Chemical analysis of leaf photosynthetic pigments and volatile apocarotenoids

Photosynthetic pigments of leaves were analysed using the method described in Lashbrooke *et al.*[43].

Leaf volatiles were extracted according to the method described by Lücker *et al.*[40] with modifications. Frozen, ground leaf tissue (200 mg) was placed in a 20 mL SPME vial and 10 mL of 20% (w/v) NaCl containing 160 ng 3-octanol (as internal standard, IS). The vial was sealed with a PTFE/silicon septum. Samples in sealed SPME vials were heated to 80°C and incubated for 5 minutes before the injection needle was pierced through the septum exposing the divinylbenzene/Carboxen/polydimethylsiloxane (DVB/CAR/PDMS) 50/30 μm coated solid phase microextraction (SPME) fibre (Supelco, Belfonte, PA, USA) to the headspace of the sample. During extraction the sample was stirred at 500 rpm and maintained at 80°C. After 15 minutes the fibre was removed and injected into the GC inlet where it was desorbed for 10 minutes at 260°C.

Extracts were analysed using an Agilent 6890 Gas Chromatograph coupled to a Waters GCT Time of Flight (TOF) Mass Spectrometer (MS) (Waters Corporation, Milford, MA, USA) Waters Masslynx GC/MS workstation software were used to analyse the data. The injector port was heated to 260°C and splitless injection (with a purge time of 3 minutes) was used. Separation was performed on an HP5MS (Agilent Technologies, Palo Alto, CA) column (30 mL × 0.25 mm i.d. × 0.25 μm f.t.) with helium as the carrier gas at a constant flow of 1 mL.min^-1^. The initial oven temperature was 40°C for 5 minutes, after which the temperature was increased by 5°C.min^-1^ to 150°C and then at 10°C.min^-1^ to 280°C (held for 2 minutes). Ionisation was in electron impact mode with an electron energy of 70 eV. The MS detector was set as follows: The transfer line, ion source and trap temperatures were 250, 180 and 150°C, respectively. The mass range was 35 to 650 m/z, with a scan rate of 4 scans.s^-1^. Compound identification was performed by both comparisons to the retention times of authentic standards and to the NIST05 mass spectral library (National Institute of Standards, USA).

## Results

### Putative CCD-encoding gene isolation and characterisation

**Figure 1:**
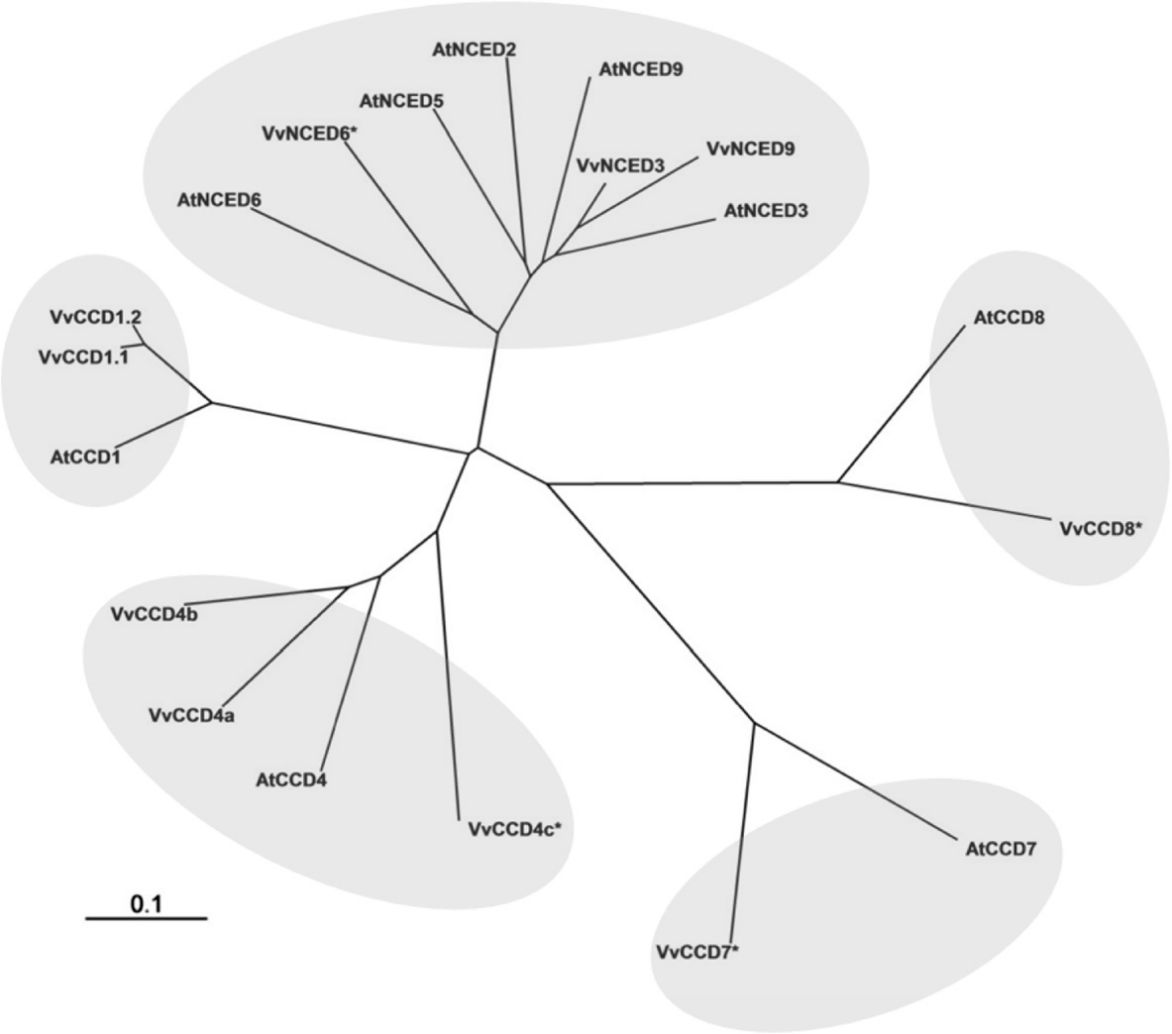
**Molecular phylogenetic analysis of *Arabidopsis thaliana *and putative *Vitis vinifera *carotenoid cleavage dioxygenases.** A Figtree generated phylogeny based on protein similarity: tree shows the various clades of the CCD family. Proteins indicated with “*” have not been isolated and are based on sequence prediction software. The CCDs are named according to their closest orthologue in *Arabidopsis thaliana*.

A phylogenetic tree of the CCD family from *A. thaliana* and *V. vinifera* and based on protein similarity is shown in Figure 1. From the molecular phylogenetic analysis five clades can be distinguished that correspond to the various orthologous groups (corresponding to the enzymatic function), namely: NCED, CCD1, CCD4, CCD7 and CCD8. In this study, three putative *VvCCD* encoding genes (*VvCCD1* [GenBank:KF008001]*, VvCCD4a* [GenBank:KF008002] and *VvCCD4b* [GenBank:KF008003]) were isolated from *V. vinifera* L. cv Pinotage cDNA using primers described in Additional file 1.

*VvCCD1* is present in the grape genome as a tandem duplication on chromosome 13 [6]. The two gene copies are situated approximately 78 kb from each other and show 96% identity at the nucleic acid level and a 97% identity on the amino acid level. Further, the 8.8 kb genomic regions of the genes (including introns and UTRs) share a 96% identity at the nucleic acid level. This suggests that the VvCCD1 isomers are functional equivalents and are treated as such in further experiments. RNAi constructs were designed to silence both isomers while real time primers did not distinguish between the two transcripts. Southern blot analysis of *VvCCD1* confirmed that *V. vinifera* cv. Sultana genomic DNA possesses two copies of the gene (Additional file 3). According to the genome sequence *CCD4a* and *CCD4b* are within 26 kb of each other on chromosome 2; both genomic copies consist of a single exon (i.e. no introns) and code for 599 aa and 589 aa proteins, respectively, sharing 70% identity on amino acid level.

An additional CCD4 orthologue (*VvCCD4c*) was identified through sequence similarity and was localised to chromosome 16, however expression of this orthologue could not be detected in any of the tissues analysed and it was therefore not isolated.

*In silico* protein localisation predicted a cytosolic localisation for VvCCD1 and a plastidial localisation for VvCCD4a and VvCCD4b (Additional file 4). Protein alignments between CCD orthologues display a highly conserved amino acid sequence (Additional file 5). Four crucial histidine residues that are conserved in CCD orthologues were identified in *VvCCD1*, *VvCCD4a* and *VvCCD4b* (Additional file 4 and Additional file 5) [44]. These amino acids are involved in the binding of an iron cation (Fe^2+^) which has been shown to be a co-factor for the carotenoid cleavage reaction [45].

### Functionality of the putative VvCCD enzymes in *Escherichia coli*

To determine the enzymatic function of the isolated putative CCD-encoding genes; the coding regions were cloned into an *E. coli* expression vector (pTWIN1). The recombinant proteins were co-expressed in carotenoid accumulating *E. coli* strains engineered to accumulate specific carotenoids: phytoene (via pAC-PHYT), ζ-carotene (via pAC-ZETA), neurosporene (via pAC-NEUR), lycopene (via pAC-LYC), ϵ-carotene (via pAC-EPSILON) and β-carotene (via pAC-BETA) strains as described in Cunningham *et al.*[32,33]. Where possible, carotenoid production in the strains used was verified using UPLC (Additional file 6). Enzyme activities were monitored after induction of the recombinant CCD proteins in the carotenoid-accumulating strains by measuring the formation of the volatile apocarotenoid cleavage products via headspace (HS)-SPME GC/MS.

**Figure 2:**
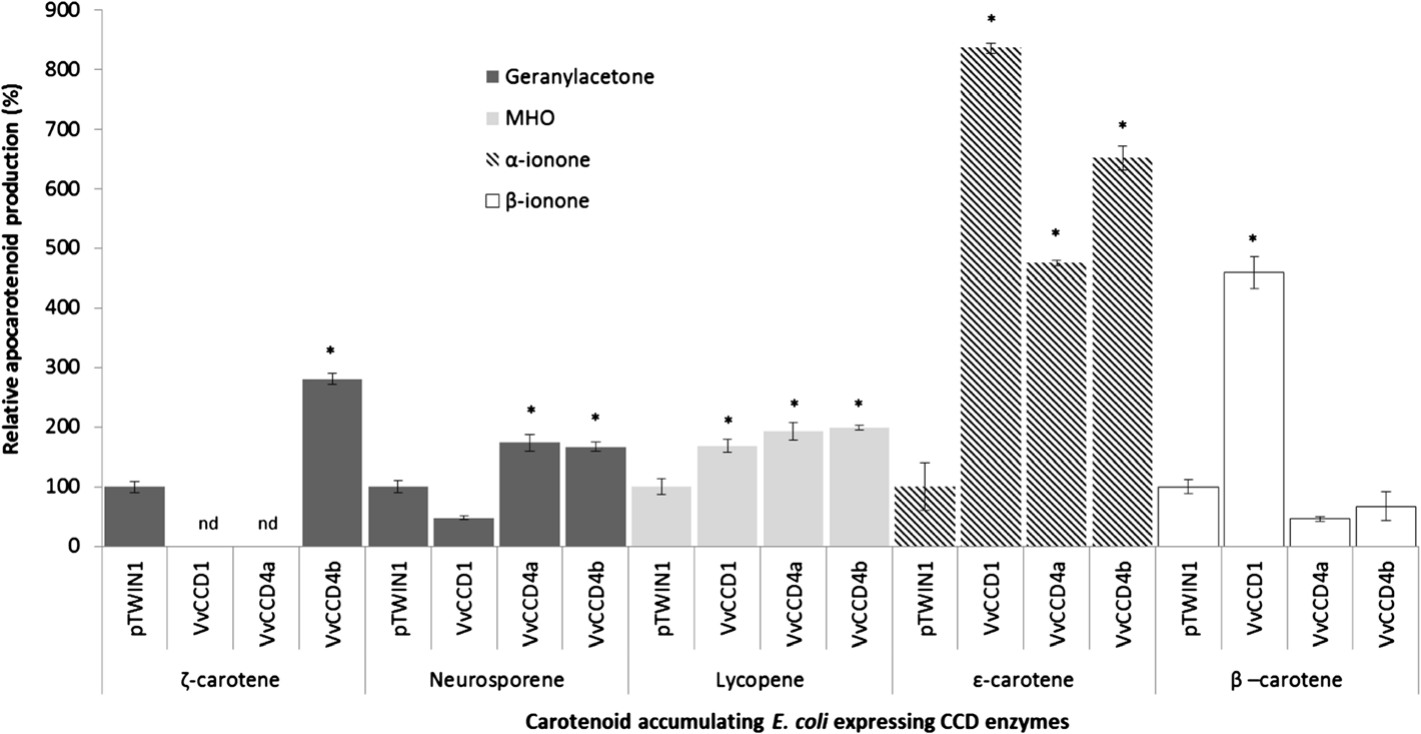
**Functionality and substrate specificity of VvCCD1, VvCCD4a and VvCCD4b in a heterologous *in vivo *bacterial system.** CCDs were expressed in *Escherichia coli* engineered to accumulate specific carotenoids. Volatile apocarotenoids produced after cleavage were determined using GC/MS. Data is represented as the average and standard deviation of three biological repeats (n = 3). pTWIN1 represents the empty vector control. Significant differences between pTWIN1 (control) and VvCCDs are indicated with an asterisk (* = *p*-value ≤ 0.01). nd = not determined.

The data, as presented in Figure 2 and Additional file 6, shows that VvCCD1, VvCCD4a and VvCCD4b are functional and have unique substrate preferences/specificities. VvCCD1, VvCCD4a and VvCCD4b all catalysed lycopene cleavage (to form 6-methyl-5-hepten-2-one; MHO) and ϵ-carotene (to form α-ionone). Neurosporene cleavage was catalysed by only VvCCD4a and VvCCD4b (not by VvCCD1). Only VvCCD1, however, catalysed β-carotene cleavage to form β-ionone; and only VvCCD4b catalysed ζ-carotene cleavage to form geranyl acetone. None of the three CCDs demonstrated an ability to catalyse the cleavage of phytoene. The production of α-ionone from ϵ-carotene provides evidence that VvCCD1, VvCCD4a and VvCCD4b possess the ability to catalyse 9,10(9’,10’) cleavage, whereas the formation of 6-methyl-5-hepten-2-one from lycopene demonstrates additional catalysis of 5,6(5’,6’) cleavage.

### Spatial and temporal expression of CCDs in grapevine organs

**Figure 3:**
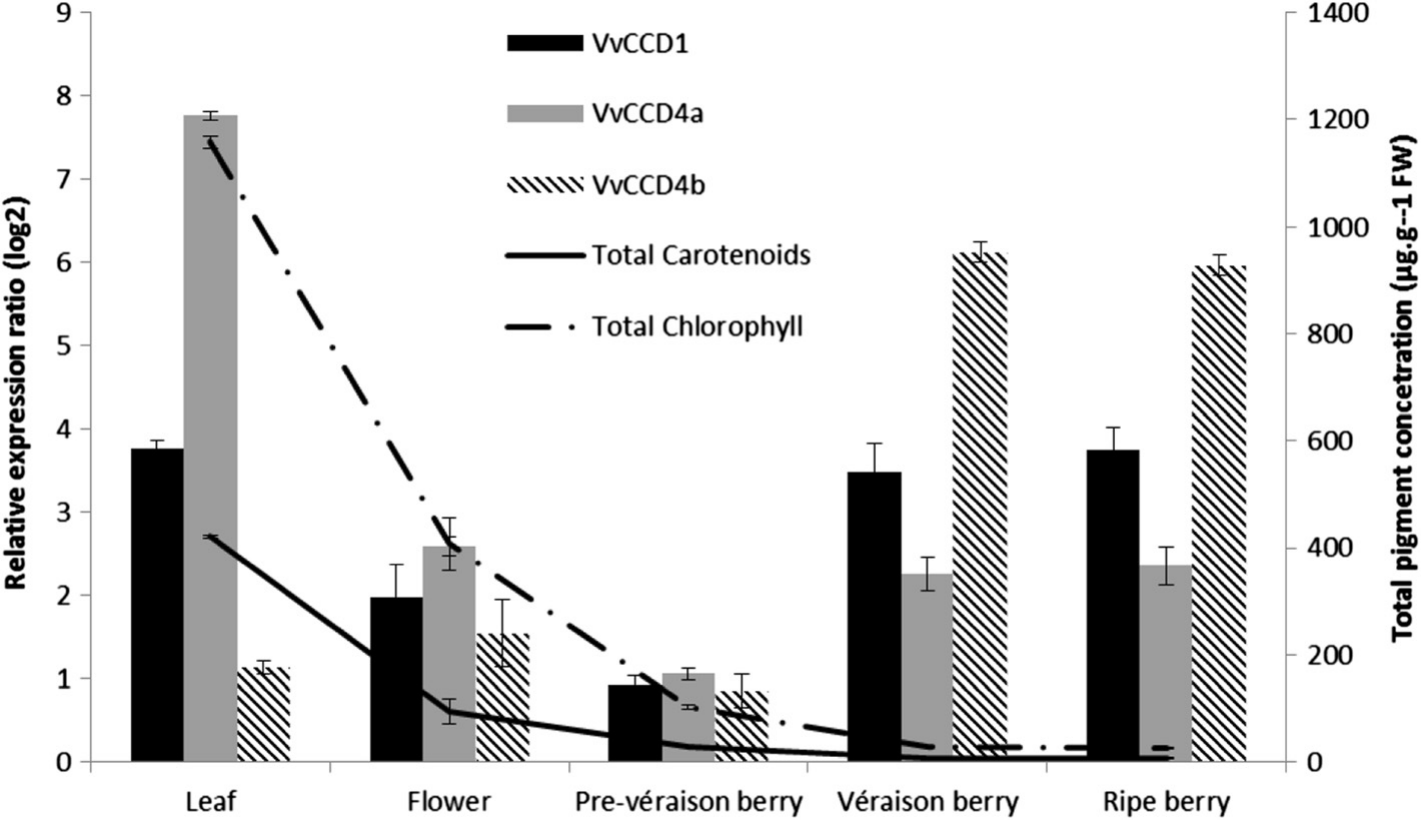
**Spatial and temporal distribution of *VvCCD1*, *VvCCD4a *and *VvCCD4b *transcripts in distinct grapevine tissues.** qRT-PCR analysis of *VvCCD1*, *VvCCD4a* and *VvCCD4b* in leaf, flower and three berry developmental stages. Data are expressed relative to pre-véraison berry stage and normalised to the housekeeping gene, *VvEF1a*. Relative changes in the total carotenoid and chlorophyll concentrations in the respective tissues are also shown.

The expression levels of *VvCCD1*, *VvCCD4a*, *VvCCD4b, VvCCD4c, VvCCD7* and *VvCCD8* were analysed in leaves, flowers and three grape berry developmental stages: green, véraison and ripe (Figure 3 and Additional file 7). No expression could be detected for *VvCCD4c, VvCCD7* or *VvCCD8* in any of the tissues or developmental stages tested using a qRT-PCR assay (results not shown), and therefore only expression of *VvCCD1*, -*4a* and -*4b* are reported and discussed further. The data shows that the *VvCCDs* were expressed in all tissues analysed. Specific patterns were, however, evident in the expression profiles: *VvCCD1* expression appeared constitutive with the highest relative levels in leaves. *VvCCD4a* was most abundant in leaves; whereas *VvCCD4b* expression was highest in berries. *VvCCD1*, *VvCCD4a* and *VvCCD4b* were all expressed in flower tissue at relatively low levels.

The lowest relative expression levels for *VvCCD1*, *VvCCD4a* and *VvCCD4b* were all in young (green) berries. The highest *VvCCD1* and *VvCCD4a* expression levels were in leaf tissue; whereas the highest expression for *VvCCD4b* was in ripe berries (Figure 3 and Additional file 7). *VvCCD1*, *VvCCD4a* and *VvCCD4b* expression all increased with development (ripening) in the berry stages. *VvCCD1* and *VvCCD4a* expression peaked and levelled off at véraison, whereas *VvCCD4b* expression increased dramatically throughout berry ripening.

### Transgenic manipulation of VvCCD1 levels

Transformation of *V vinifera* L. cv Sultana with a pART27-VvCCD1 overexpression cassette, was confirmed by Southern hybridisation (Additional file 3)*.* Digestion of genomic DNA with *Spe*I resulted in a single hybridisation band per integration event as the restriction enzyme digests outside of the zone of hybridisation. Analysis of the transgenic plants showed that six of the nine lines represented independent integration events, with a transgene copy number ranging between one and four. The CCD1-10 and CCD1-12 lines, as well as the CCD1-15, CCD1-17, and CCD1-19 lines were considered clonal (Additional file 3). Twelve lines were shown to be positive for transformation/integration of the transgene with a silencing cassette via PCR screening (data not shown).

**Figure 4:**
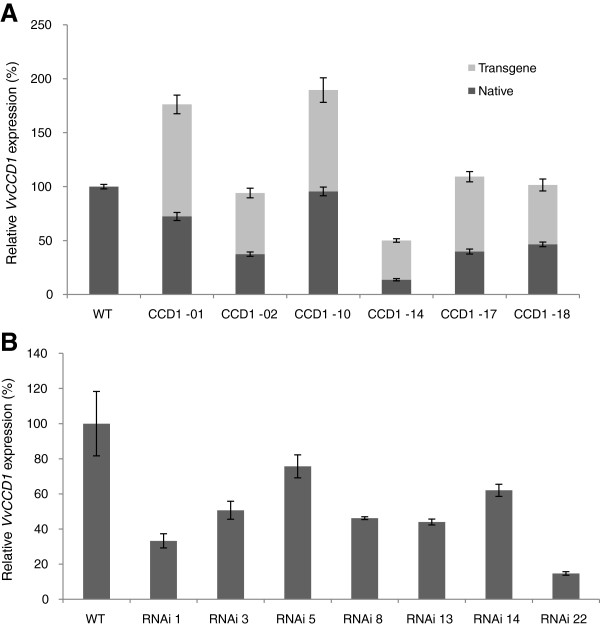
**qRT-PCR analysis of *VvCCD1 *expression in the transgenic grapevine population. (A)** Expression of the native/endogenous (dark grey square symbol) and transgenic (light grey square symbol) *VvCCD1* in lines transformed with the overexpression cassette (CCD1) (n = 3). **(B)** Expression of *VvCCD1* in lines transformed with the silencing cassette (RNAi) (n = 3). Data are expressed relative to the wild-type (WT) expression and normalised to *VvGAPDH* expression.

The level of *VvCCD1* expression in the transgenic lines was monitored via qRT-PCR. Of the six lines independently transformed with pART27-VvCCD1, only two lines showed significant overexpression of *VvCCD1* (up to an 85% increase relative to the expression seen in wild-type) (Figure 4). qRT-PCR expression analysis demonstrated transcription of the endogenous gene as well as the introduced transgene in all the lines, yet four lines displayed total *VvCCD1* gene expression levels which were not significantly increased (when compared to the wild-type) (Figure 4). It is interesting to note that the transgene-derived expression was fairly constant whereas the endogenous gene expression was more affected. Of the twelve lines positively transformed with pART27-CCD1(RNAi), seven showed significant silencing when compared to the wild-type lines (Figure 4). Silencing of *VvCCD1* of up to 85% (relative to wild-type expression) was observed. Targeted metabolite analyses were performed on plant lines with expression levels that differed significantly from the wild-type to determine if the carotenoid (i.e. the cleavage substrates) and/or the volatile apocarotenoid profiles (i.e. cleavage products) were affected/altered.

### Carotenoid, chlorophyll and apocarotenoid analysis of transgenic grapevine lines

**Figure 5:**
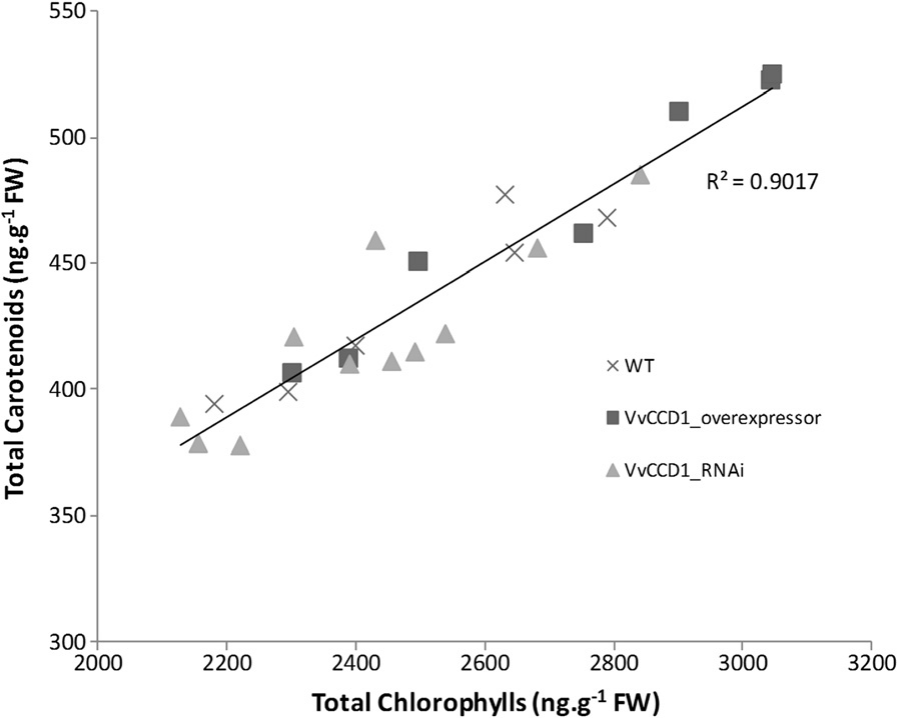
**The relationship between carotenoids and chlorophylls in the leaves of the grapevine population altered for *VvCCD1 *expression.** RP-HPLC analysis of the *VvCCD1*-overexpressing (dark grey square symbol), *VvCCD1*-silenced (RNAi) (light grey triangle symbol) and wild-type (×) grapevine plants used in the study showed positive correlation (R^2^ = 0.90) between the total carotenoid concentration and the total chlorophyll concentration in the leaves (n = 3).

RP-HPLC analysis of pigments extracted from the transgenic population showed no significant correlation between *VvCCD1* expression levels and the concentration of carotenoids in grapevine leaf tissue under the conditions tested (Additional file 8). Similarly, no correlation between *VvCCD1* expression and leaf norisoprenoid levels were observed in the same tissue (Additional file 9). Total carotenoid content however, showed strong positive correlation to the total chlorophyll content of the leaves (R^2^ = 0.90) (Figure 5).

## Discussion

### VvCCD1, VvCCD4a and VvCCD4b catalyse the cleavage of a broad range of carotenoid substrates

**Figure 6:**
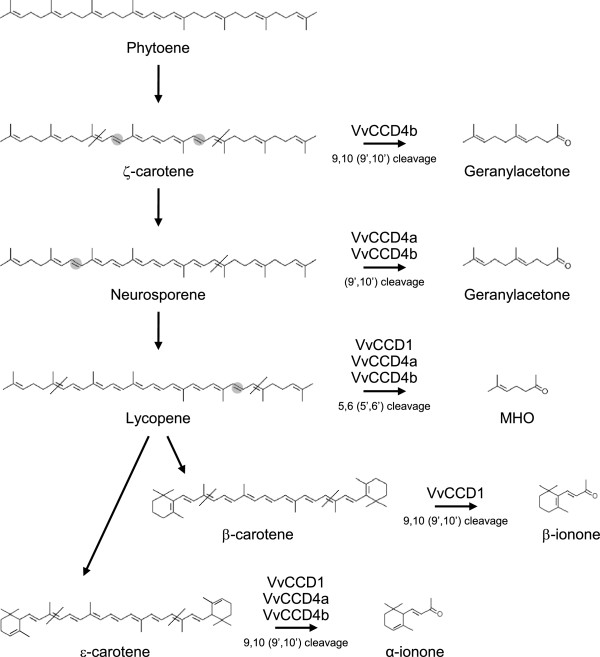
**VvCCD-mediated cleavage of members of the carotenoid biosynthetic pathway.**The cleavage of tested members of the carotenoid biosynthetic pathway is shown together with the volatile apocarotenoids produced. The 5,6 (5’,6’) and 9,10 (9’,10’) double bond cleavage sites are indicated with a diagonal line (/) through the carotenoid backbone. The circled (light grey circle) double bonds represent an increase in desaturation of the carbon bonds that is required for substrate acceptance.

The enzymatic catalysation of the cleavage of carotenoids by CCDs has been shown to produce volatile flavour and aroma apocarotenoids including α-ionone, β-ionone and 6-methyl-5-hepten-2-one (MHO) in a range of plant species. Apocarotenoids contribute to the floral and fruity attributes of many wine cultivars and are thereby partly responsible for the “varietal character” of grapes and wine (e.g. Chenin blanc, Semillon, Sauvignon blanc, Cabernet Sauvignon, and Shiraz). Here we confirmed the functionality of VvCCD1, and for the first time VvCCD4a and VvCCD4b on a range of carotenoid substrates. The identification of CCD4 orthologues from grapevine has recently been reported [6,38]; and Guillaumie *et al.*[38] tested a CCD4a isolated from *V. vinifera* cv Chardonnay on ζ-carotene, lycopene, β-carotene, and zeaxanthin, but could not show functionality on any of the substrates tested. CCD1 and CCD4 orthologues in other plant species have been shown to catalyse cleavage of a number of C_40_-carotenoid and C_30_-apocarotenoid substrates. Here we show VvCCD1, VvCCD4a and VvCCD4b are capable of catalysis of the cleavage of C_40_ carotenoid substrates at the 9, 10 (9’, 10’) (ζ-carotene, β-carotene and/or ϵ-carotene) and lycopene at the 5, 6 (5’, 6’) double bond position to release the corresponding C_13_ apocarotenoid products (Table 1 and Figure 6), confirming cleavage of both cyclic and linear substrates. This is the first example of a CCD4 enzyme catalysing the cleavage of linear carotenoids and is in contrast to the conclusions made by Huang *et al.*[11] who, after analysing the functionality of CCD4s from five different plant species (excluding grapevine) concluded that CCD4s catalyse the cleavage of cyclic non-polar carotenoids (e.g. β-carotene) and not linear carotenoids (e.g. lycopene or ζ-carotene). Interestingly, only VvCCD4b catalysed the cleavage of ζ-carotene and neither VvCCD4a nor VvCCD4b could catalyse β-carotene cleavage. None of the CCDs tested catalysed the cleavage of phytoene. These results are in agreement with the hypothesis by Vogel *et al.*[25], who suggest that CCD-catalysed cleavage only occurs when a double bond is found adjacent to the bond that is to be cleaved. In the case of 5, 6 (5’, 6’) or 9, 10 (9’, 10’) cleavage a double bond must therefore be found at the 7, 8 (7’, 8’) or 11, 12 (11’, 12’) positions, respectively. Phytoene does not possess these double bonds and is therefore not cleaved. The ability of VvCCD4b to catalyse the cleavage of ζ-carotene to form geranylacetone, but not MHO, further illustrates this point (Figure 6). Carotenoids containing hydroxyl groups (e.g. lutein and zeaxanthin) as potential substrates for the VvCCD4 catalysed cleavage were not investigated in our study and remains to be evaluated.

**Table 1:** A summary of plant CCDs identified, including the grapevine enzymes, with cleavage sites, substrates and products

**Species**	**Enzyme**	**Cleavage site**	**Substrates**	**Products**	**Reference**
*Arabidopsis thaliana* (Thale cress)	AtCCD1	9,10 (9’,10’)	β-carotene	β-ionone	[46]
		9,10 (9’,10’)	zeaxanthin	3-hydroxy-β-ionone	[47]
		9,10 (9’,10’)	lutein	3-hydroxy-β-ionone	[25]
		9,10 (9’,10’)	violaxanthin	5'6-epoxy-3-hydroxy-b-ionone	
		9,10 (9’,10’)	neoxanthin	5'6-epoxy-3-hydroxy-b-ionone	
		9,10 (9’,10’)	lycopene	pseudoionione	
		5,6 (5’,6’)	lycopene	MHO	
	AtCCD4	9,10 (9’,10’)	8’-apo-β-caroten-8’-al	β-ionone	[11]
*Solanum lycopersicon* (previously *Lycopersicon esculentum*) (Tomato)	LeCCD1a	9,10 (9’,10’)	lutein	3-hydroxy-b-ionone	[18]
		9,10 (9’,10’)	violaxanthin	5'6-epoxy-3-hydroxy-b-ionone	[25]
		9,10 (9’,10’)	neoxanthin	5'6-epoxy-3-hydroxy-b-ionone	
		9,10 (9’,10’)	lycopene	pseudoionone	
		5,6 (5’,6’)	lycopene	MHO	
		9,10 (9’,10’)	β-carotene	β-ionone	
		9,10 (9’,10’)	zeaxanthin	3-hydroxy-β-ionone	
	LeCCD1b	9,10 (9’,10’)	β-carotene	β-ionone	[25]
		9,10 (9’,10’)	lycopene	pseudoionone	
		5,6 (5’,6’)	lycopene	MHO	
*Petunia hybrid* (Petunia)	PhCCD1	9,10 (9’,10’)	β-carotene	β-ionone	[18]
*Cucumis melo* (Musk melon)	CmCCD1	9,10 (9’,10’)	phytoene	geranylacetone	[48]
		5,6 (5’,6’)	lycopene	pseudoionone	
		9,10 (9’,10’)	β-carotene	β-ionone	
		9,10 (9’,10’)	δ-carotene	α-ionone and pseudoionone	
*Vitis vinifera* (Grapevine)	VvCCD1	9,10 (9’,10’)	zeaxanthin	3-hydroxy-β-ionone	[54]
		9,10 (9’,10’)	lutein	3-hydroxy-β-ionone	[49]
		5,6 (5’,6’)	lycopene	MHO	This study
		9,10 (9’,10’)	ϵ-carotene	α-ionone	
	VvCCD4a	9,10 (9’,10’)	ϵ-carotene	α-ionone	This study
		9,10 (9’,10’)	neurosporene	geranylacetone	
		5,6 (5’,6’)	lycopene	MHO	
	VvCCD4b	9,10 (9’,10’)	ϵ-carotene	α-ionone	This study
		9,10 (9’,10’)	neurosporene	geranylacetone	
		9,10 (9’,10’)	ζ-carotene	geranylacetone	
*Crocus sativus* (Saffron crocus)	CsCCD1a	9,10 (9’,10’)	β-carotene	β-ionone	[50]
		9,10 (9’,10’)	zeaxanthin	3-hydroxy-β-ionone	[23]
	CsCCD1b	9,10 (9’,10’)	β-carotene	β-ionone	[50]
		9,10 (9’,10’)	zeaxanthin	3-hydroxy-β-ionone	[23]
	CsCCD4a	9,10 (9’,10’)	β-carotene	β-ionone	[23]
	CsCCD4b	9,10 (9’,10’)	β-carotene	β-ionone	[22]
	CsZCD	7,8(7′,8′)	zeaxanthin	crocetin dialdehyde	[50]
*Fragaria ananassa* (Strawberry)	FaCCD1	9,10 (9’,10’)	zeaxanthin	β-ionone	[51]
		9,10 (9’,10’)	lutein	3-hydroxy-β-ionone	
		9,10 (9’,10’)	β-apo-8’-carotenol	3-hydroxy-a-ionone	
*Zea mays* (Maize)	ZmCCD1	9,10 (9’,10’)	ζ-carotene	geranylacetone	[25]
		9,10 (9’,10’)	lycopene	pseudoionone	
		5,6 (5’,6’)	lycopene	MHO	
		9,10 (9’,10’)	δ-carotene	α-ionone	
		9,10 (9’,10’)	β-carotene	β-ionone	
		9,10 (9’,10’)	zeaxanthin	3-hydroxy-β-ionone	
*Rosa damascena*(Damask rose)	RdCCD1	9,10 (9’,10’)	β-carotene	β-ionone	[11]
		9,10 (9’,10’)	ζ-carotene	geranylacetone	
		9,10 (9’,10’)	neoxanthin	grasshopper ketone	
		9,10 (9’,10’)	lycopene	pseudoionone	
		5,6 (5’,6’)	lycopene	MHO	
		9,10 (9’,10’)	zeaxanthin	3-hydroxy-β-ionone	
	RdCCD4	9,10 (9’,10’)	β-carotene	β-ionone	[11]
		9,10 (9’,10’)	8’-apo-β-caroten-8’-al	β-ionone	
*Oryza sativa* (Asian rice)	OsCCD1	5,6 (5’,6’)	Lycopene	MHO	[20]
		7,8 (7’,8’)	lycopene	geranial	
		9,10 (9’,10’)	lycopene	pseudoionone	
*Osmanthus fragrans* (Sweet osmanthus)	OfCCD1	9,10 (9’,10’)	α-carotene	α-ionone	[42]
			β-carotene	β-ionone	
*Chrysanthemum morifolium*(Chrysanthemum)	CmCCD4a	9,10 (9’,10’)	β-carotene	β-ionone	[11,52]
*Malus domestica* (Apple)	MdCCD4	9,10 (9’,10’)	β-carotene	β-ionone	[11]
*Solanum tuberosum* (Potato)	StCCD4		Unknown carotenoid	MHO	[53]

Mathieu *et al.*[54] used an *in vitro* enzyme assay to demonstrate that a VvCCD1 from *V. vinifera* L. cv Shiraz catalysed the cleavage of the xanthophylls zeaxanthin and lutein to form 3-hydroxy-β-ionone. The authors stated that β-carotene was tested as a substrate, but that it was not cleaved in their assay. VvCCD1 isolated from *V. vinifera* L. cv Pinotage in this study, however, was capable of catalysing the cleavage of lycopene, β-carotene and ϵ-carotene, but not neurosporene and ζ-carotene (Figure 2 and Figure 6). Cross-comparing results from different studies are often complicated by the differences in the assays utilised for experiments of this nature (i.e. *in vivo* versus *in vitro* enzyme assays) and/or the choice of analytical methods used to generate the data (i.e. degradation of carotenoids versus formation of apocarotenoids). One of the advantages of the *in vivo* assay used in this study is the controlling and validation of the specific carotenoid substrates. Carotenoid biosynthesis in the *E. coli* strains was controlled by the addition of the carotenoid pathway inhibitor diphenylamine (DPA; according to Cunningham and Gantt [40]) whereas the carotenoid(s) produced in the respective strains after 2 hours of DPA inhibition were verified by UPLC before induction of the respective CCDs. Steps were further taken to prevent the oxidative, non-enzymatic degradation of the carotenoids and a co-factor was added to ensure functionality of the CCD enzymes in the *in vivo* assays (according to Baldermann *et al*. [42]).

### *In vivo* functions of the isolated VvCCDs

#### VvCCD1, VvCCD4a and VvCCD4b influence the flavour and aroma potential of grapes

*VvCCD1, -4a* and *4b,* were all up-regulated during ripening (Figure 3), and are therefore all potentially involved in the enzymatic degradation of carotenoids to the respective aromatic C_13_-norisoprenoids that contribute to the distinctive varietal character of grapes and wine. Since the *VvCCD1, -4a* and *-4b* are all expressed during ripening, the specific carotenoid substrate(s) available for cleavage, as well as the substrate specificity of the respective VvCCD(s) present, will determine the C_13_-norisoprenoid(s) formed in grapes. Although *VvCCD1*, *VvCCD4a* and *VvCCD4b* are expressed during berry development; *VvCCD4b* showed the most berry-specific expression profile and the highest upregulation throughout berry ripening (30-fold upregulation at the ripe stage relative to the green stage) and appears to be the isoform most likely responsible for catalysing carotenoid cleavage in ripening berries. *VvCCD4a* showed the highest expression in leaves and *VvCCD1* maintains relatively constant levels in all tissues tested. In agreement with the expression data presented here, Mathieu *et al.*[54] showed similar trends for *VvCCD1* expression in berries (i.e. peaking at véraison), and, while the authors noted an increase in the C_13_-norisoprenoid content of Muscat (and to a lesser extent Shiraz), this occurred two weeks after the increase in *VvCCD1* expression. Numerous studies on grape berry carotenoid composition have shown that carotenoid concentrations decrease during ripening [14,43,55]. The inverse correlation observed between carotenoids and C_13_-norisoprenoids lead Crupi *et al.*[15] to propose that the change in carotenoid concentration in grape berries from véraison stage to the ripe (harvest) stages could be used to estimate the aromatic potential of the grapes.

#### VvCCD4a and VvCCD4b are candidates for maintenance of carotenoid levels during photosynthesis

The relative abundance of oxygenated carotenoids (xanthophylls) is conserved and tightly regulated in photosynthetic tissues and is largely due to the involvement of specific pigments in the photosynthetic reaction centres of Photosystems (PS) I and II (mainly β-carotene) and the light harvesting antennae complexes (mainly the xanthophylls lutein, violaxanthin, and neoxanthin).

Although carotenoid composition is considered highly conserved in photosynthetic organisms with the ubiquitous presence of the xanthophylls lutein, neoxanthin and violaxanthin most notable; mutant studies in *A. thaliana* have shown that a certain degree of plasticity is tolerated in plants. Plants have been shown to be viable even in the absence of lutein, neoxanthin and violaxanthin, but these xanthophylls are required for optimal seedling development and photoprotection, and surprisingly to a lesser extent photosynthesis [56].

The VvCCD1 transgenic lines also showed positive correlation between the concentrations of chlorophyll and carotenoids in mature photosynthetically active leaves (Figure 5; R^2^ = 0.90). This is expected as carotenoids are primarily involved in photosynthesis specifically in vegetative tissue [4]. An increase in chlorophyll concentration is indicative of increased photosynthetic activity, which in turn puts more demand on the carotenoids involved in light-harvesting and photoprotection [57]. It is therefore not surprising that the carotenoid pathway is tightly regulated in leaf tissue, as loss of these crucial pigments would lead to an impaired and consequently sub-optimal plant [58-61].

Beisel *et al.*[62] demonstrated continuous synthesis and degradation of carotenoids and chlorophyll a in mature leaves of *Arabidopsis*. This turnover of carotenoids and chlorophylls appears crucial for the maintenance of active photosynthesis and for adaptation to changing light conditions. It is possible that the CCDs (especially chloroplast localised CCD4 orthologues) are involved in the maintenance of the optimal carotenoid composition in photosynthetic tissue. Static plants confronted with an ever-changing environment must be capable of maintaining an optimal carotenoid composition in photosynthetic tissues. Due to their localisation and substrate specificities, CCDs may be involved in this regulation and maintenance. We show that the cytosolic VvCCD1 catalyses lycopene, β-carotene and ϵ-carotene (but not ζ-carotene) cleavage (Figure 2), whereas the chloroplast localised VvCCD4a and VvCCD4b catalyses lycopene, neurosporene and ϵ-carotene (but not β-carotene) cleavage (Figure 2). Only VvCCD4b was able to catalyse ζ-carotene cleavage. Of these carotenoid substrates, only β-carotene is present at detectable levels in grapevine leaves and berries (Figure 3 and Additional file 7), where it forms part of photosystem I and –II. Differential transcriptional regulation and the physical separation of the carotenoid substrates (chloroplastic) from the VvCCD1 enzyme (cytosolic) therefore add additional layers of possible control.

#### Altering the expression of *VvCCD1* in transgenic grapevine suggests sub-cellular compartmentalisation regulates CCD activity

Our transgenic data confirmed a level of protection from manipulation of the carotenoid pathway, specifically from altered levels of *VvCCD1*. Significant overexpression of the *VvCCD1* was only observed in 30% of the Southern positive grapevine lines. This was not due to a non-functional construct, since qRT-PCR experiments verified endogenous and the transgenic *VvCCD1* expression in the plant lines (Figure 4). In spite of the partial silencing of most of the overexpression lines (by controlling the endogenous gene expression level); lines were generated that exhibited varying degrees of overexpression of *VvCCD1* (Figure 4). The overexpressing lines, together with the lines that showed successful silencing, resulted in a population of transgenic plants that exhibited an up to ~12-fold range of *VvCCD1* expression (from the most silenced line to the most overexpressed line).

Despite this range in *VvCCD1* expression no direct phenotype was observed in the vegetative (leaf) tissue tested for either the carotenoids (substrates) or apocarotenoids (products) based on known VvCCD1 enzyme activity. Mathieu *et al.*[54] monitored ripening berries and found a similar discrepancy (or lack of correlation) between *VvCCD1* expression and apocarotenoid production. Since quantification of the VvCCD1 protein was not performed on the transgenic lines, it is not possible to speculate on the relationship between *VvCCD1* transcripts and VvCCD1 protein, but it is known that a positive correlation between mRNA levels and protein levels is far less common than often assumed [63]. Another factor that could contribute to the lack of an observable phenotype in the leaf tissue analysed, is the compartmentalisation of the enzymes within a plant cell. *In silico* analysis suggest that VvCCD1 is localised in the cytosol. This has been shown to be the case for many CCD1 orthologues [25]. The carotenoid cleavage substrates that CCD1 has been shown to catalyse *in vitro*, however, are situated in the chloroplast membrane, and are thus inaccessible to the CCD1 enzyme [4].

Previous attempts to manipulate the expression levels of *CCD1* transcripts have also led to phenotypes inconsistent with the observed *in vitro* activity of CCD1 [18,19]. Simkin *et al.*[18] reduced *LeCCD1* mRNA levels by up to 90% in tomato leaves and fruit using an antisense construct, but did not observe any change in the carotenoid concentration in fruit with reduced *CCD1* levels, and only a 50% decrease in the β-ionone concentration in selected silenced lines. No data was presented for carotenoid or norisoprenoid levels in tomato leaves with reduced *CCD1* levels. Ilg *et al.*[20] suggested that *in planta* OsCCD1 may catalyse apocarotenoid cleavage rather than carotenoids. A similar reduction of the norisoprenoids observed in tomato fruit with reduced *CCD1* transcripts was not seen in grapevine leaves (Additional file 9). These studies, and supported by the results presented in this study, suggest that the *in planta* role of CCD1 may in fact not be maintenance of carotenoid levels, but catalysing apocarotenoid cleavage. It is not surprising when considering that the leaf is the primary photosynthetic organ of the plant and therefore needs to maintain this function. The correlation observed between chlorophyll concentration and carotenoid concentration in the leaf (Figure 5) indicates that the level of photosynthetic activity of the tissue has a much greater influence on the concentration of carotenoids. In the chromoplasts of ripe fruit the need for photoprotection of the chlorophylls is absent and the segregation of CCD1 and its carotenoid substrates is possibly diminished. However, despite the predicted cytosolic localisation of CCD1 and its apparent lack of transcript correlation with carotenoid and norisoprenoid concentrations, norisoprenoids are still formed in the leaves (Additional file 9). This suggests another mechanism of action for the cleavage of carotenoids and the production of norisoprenoids. The CCD4s are the most likely candidates due to the fact that the *VvCCD4a* and *VvCCD4b* transcripts are upregulated during berry development (peaking at harvest for *VvCCD4b*); subcellularly both VvCCD4a and VvCCD4b are predicted to co-localise with carotenoids in the chloroplast; and the catalysis of carotenoid cleavage by VvCCD4a and VvCCD4b have been demonstrated.

On a sub-organellar level, a number of carotenoid metabolic enzymes have been identified in the plastoglobule proteome of *A. thaliana*, including CCD1 and CCD4. Interestingly the CCD4 protein represented 3.3% of the total mass of the plastoglobule proteome [64]. Plastoglobules are thylakoid-associated lipoprotein particles found in plastids (i.e. suborganellar compartmentalisation). It is thought that lipid exchange (i.e. carotenoids, plastoquinones, tocopherols) can occur between the plastoglobules and the thylakoid membrane. The size and abundance of plastoglobules in chloroplasts are affected by a number of developmental and environmental conditions, and includes stresses (especially oxidative) and transitions from chloroplasts to gerontoplasts (i.e. senescence) and chloroplast to chromoplast transitions (e.g. as occurs in flowers and fruit).

Plastids can differentiate and dedifferentiate and the size and number of plastoglobules increases during these transitions. It is interesting to note that CCD1, CCD4 and CCD8 have been localised to plastoglobules; and that the carotenoid substrates accumulate, and can be formed *de novo* in plastoglobules. The *in planta* role of the CCDs in these lipoprotein structures is still not clear. If they are required to maintain the carotenoid composition and/or it is for the production of the apocarotenoid cleavage product that drives carotenoid cleavage remains to be elucidated.

## Conclusions

To summarise, VvCCD1 in grapevine leaves appears to be under various levels of control. All factors being considered, the importance of the carotenoid composition for effective photosynthesis is the most likely reason for this control in the leaf. The control is applied at the transcript level, where a form of post-transcriptional gene silencing was observed in the transgenic VvCCD1 population; and possibly on the protein level, where sub-cellular compartmentalisation may prevent interaction between VvCCD1 and its carotenoid substrate(s). The substrate specificity of the respective VvCCDs characterised in this study (i.e. VvCCD1, VvCCD4a and VvCCD4b), suggest another level where control can be exerted. Despite the fact that VvCCD1 has the ability to catalyse the cleavage of multiple carotenoids *in vitro*, the *in planta* substrates for cleavage may be primarily C_27_ apocarotenoids produced through cleavage by enzymatic action (by CCD4 and/or CCD7 as suggested by Floss *et al.*[19] or photo-oxidation, and subsequently transported from the chloroplast to the cytosol. In either case grape berries would likely display a phenotype more closely correlated to *VvCCD1* expression due to the increased osmotic stress that occurs during ripening, resulting in leaky membranes and the concomitant degradation of chloroplasts. The extensive youth phase/maturation period in grapevine (a woody perennial) that can last in excess of three years, excluded the analysis of berries from the transgenic populations generated from the scope of this study.

The isolation and functional characterisation of VvCCD4a and VvCCD4b provides additional candidate cleavage enzymes affecting the carotenoid composition in the chloroplast, as well as the production of volatile apocarotenoids in grapevine. Their differential expression in various plant tissues and the differential substrate specificities of the VvCCD1, VvCCD4a and VvCCD4b suggests that CCDs have distinct roles in different plants, plant tissues and even different subcellular compartments of plants (i.e. plastids). Future studies on, for example, the senescing leaves and grape berries from the transgenic lines generated in this study will be of great importance in further elucidating the *in planta* function of CCDs. Generation of transgenic grapevine altered in *VvCCD4* expression as well as future studies on the senescing leaves and grape berries from the transgenic lines generated in this study will be of great importance in further elucidating the *in planta* function of CCDs.

## Availability of supporting data

The data sets supporting the results of this article are included within the article (and its additional files).

## Competing interests

The authors declare that they have no conflict of interest.

## Authors’ contributions

JGL, PRY and MAV conceptualised the study. JGL, PRY and MAV were involved in the experimental layout. JGL, PRY and SJD isolated and cloned the genes of interest and performed the bacterial functional complementation assays. JGL constructed the plant transformation constructs. KV performed the grapevine transformations and subsequent selection and regeneration of plantlets. JGL performed the genetic and metabolite screening of the transgenic plants. JGL, PRY and MAV drafted the initial manuscript. All authors contributed to the discussion of the results, reviewing of the manuscript and approved the final manuscript.

## Supplementary Material

Additional file 1 Primers used in the study. Click here for file

Additional file 2 Constructs and plasmids used in this study. Click here for file

Additional file 3 **Southern hybridisation confirming integration of pART27-VvCCD1.** Band sizes (in bp) of λ DNA digested with *Bst*E II (lane 1) is shown. Genomic DNA was digested with *Spe*I. Two hybridisation events in the wild-type (lane 2) indicate two copies of *VvCCD1* in the Sultana genome. Lane 3, CCD1-01; lane 4, CCD1-02; lane 5, CCD1-10; lane 6, CCD1-12; lane 7, CCD1-14; lane 8, CCD1-15; lane 9, CCD1-17; lane 10, CCD1-18; lane 11, CCD1-19. Estimated number of integration events are displayed at the bottom of each lane. Plants with the same clonal group (a-f) are considered clonal copies. Click here for file

Additional file 4 **Protein characterisation of the carotenoid cleavage dioxygenases from *A. thaliana *and the *V. vinifera *orthologues present in the grapevine genome.** Click here for file

Additional file 5 **Clustal multiple protein alignments of carotenoid cleavage dioxygenase encoding sequences of *A. thaliana *and (At-) and *V. vinifera *(Vv) orthologues.** Arrows indicate the conserved histidine residues. Click here for file

Additional file 6 **Functionality and substrate specificity of VvCCD1, VvCCD4a and VvCCD4b in a heterologous *in vivo *bacterial system.** CCDs were expressed in Escherichia coli engineered to accumulate specific carotenoids. Carotenoids produced before cleavage were determined using UPLC. Volatile apocarotenoids produced after cleavage were determined using GC-MS. Click here for file

Additional file 7 **Carotenoids and chlorophyll concentrations present in the grapevine organs investigated in this study.** Carotenoids and chlorophylls were analysed by HPLC. Individual carotenoids and chlorophylls were identified by comparison to authentic standards and quantified by normalisation to an internal standard (β-apo-carotenal) and quantified by external standard curve as described in Lashbrooke et al. (2010). Click here for file

Additional file 8 **Relationship between carotenoid concentration and *VvCCD1 *expression levels in grapevine leaves.** None of the major carotenoids in the leaves of the grapevine population (measured via HPLC) showed significant correlation with *VvCCD1* expression. The concentration of β-carotene (A), lutein (B), violaxanthin (C), neoxanthin (D), antheraxanthin (E) and zeaxanthin (F) found in wild-type (green triangle symbol), silenced (blue diamond symbol) and overexpression (red square symbol) lines is shown. Click here for file

Additional file 9 **Relationship between apocarotenoid concentration and *VvCCD1 *expression levels in grapevine leaves.** No significant correlation was found between the *VvCCD1* expression and volatile apocarotenoids in the leaves of the grapevine population (measured via GC/MS). The concentration of 6-methyl-5-hepten-2-one (MHO) (A), pseudoionone (B), α-ionone (C), β-ionone (D), and geranylacetone (E) found in wild-type (blue diamond symbol), silenced (red square symbol) and overexpression (light green triangle symbol) lines is shown. Click here for file
