# Supplementary material for: Functional characterisation of three members of the Vitis vinifera L. carotenoid cleavage dioxygenase gene family
Source: BMC Plant Biol. 2013 Oct 9;13:156. doi: 10.1186/1471-2229-13-156 (PMC3854447; doi:10.1186/1471-2229-13-156)
Supplement: Additional file 1 — Primers used in the study. [file 1471-2229-13-156-S1.pdf]

Additional file 2. Constructs and plasmids used in this study

Construct	Description
pGEMt-VvCCD1	*CCD1* PCR-amplified from *V. vinifera* cv. Pinotage cDNA using primer pair VvCCD1_5'/VvCCD1_3'and cloned into pGEM®-T Easy cloning vector.
pGEMt-VvCCD4a	*CCD4a* PCR-amplified from *V. vinifera* cv. Pinotage cDNA using primer pair VvCCD4a_5'/VvCCD4a_3'and cloned into pGEM®-T Easy cloning vector.
pGEMt-VvCCD4b	*CCD4b* PCR-amplified from *V. vinifera* cv. Pinotage cDNA using primer pair VvCCD4b_5'/VvCCD4b_3'and cloned into pGEM®-T Easy cloning vector.
pGEMt-CCD1(RNAi)	A 148 bp fragment of the 3'-untranslated region of VvCCD1 was PCR-amplified from *V. vinifera* cv. Pinotage genomic DNA using primer pair VvCCD1_RNAi_5'/ VvCCD1_RNAi_3'and cloned into pGEM®-T Easy cloning vector.
pTWIN1-VvCCD1	*VvCCD1* was isolated from pGEMt-VvCCD1 as an *Nde*I/*Pst*I fragment and cloned into the corresponding *Nde*I/*Pst*I sites of pTWIN1.
pTWIN1-VvCCD4a	*VvCCD4a* was isolated from pGEMt-VvCCD4a as an *Nde*I/*Bgl*II fragment and cloned into the compatible *Nde*I/*Bam*HI sites of pTWIN1.
pTWIN1-VvCCD4b	*VvCCD4b* was isolated from pGEMt-VvCCD4b as an *Nde*I/*Bam*HI fragment and cloned into the corresponding *Nde*I/*Bam*HI sites of pTWIN1.
pART7-VvCCD1	*VvCCD1* was isolated from pGEMt-VvCCD1 as a *Sal*I/*Spe*I fragment and cloned into the compatible *Xho*I/*Xba*I sites of pART7.
pART27-VvCCD1	The VvCCD1 expression cassette was excised from pART7 as a *Not*I fragment and cloned into the corresponding *Not*I sites of pART27.
pHANNIBAL-CCD1(RNAi)	A two-step cloning strategy was used: (1) A 136 bp *Xho*I/*Eco*RI fragment was excised from pGEMt-CCD1(RNAi) and cloned into the corresponding *Xho*I/*Eco*RI sites of pHANNIBAL; (2) a 148 bp *Bam*HI/*Xba*I fragment was excised from pGEMt-CCD1(RNAi) was cloned into the corresponding *Bam*HI/*Xba*I sites of the resultant recombinant plasmid generated in (1).
pART27-CCD1(RNAi)	The expression cassette containing the VvCCD1_RNAi inverted repeat was excised from pHANNIBAL-CCD1(RNAi) with *Not*I and cloned into the corresponding *Not*I site of pART27.
