# Supplementary material for: Functional characterisation of three members of the Vitis vinifera L. carotenoid cleavage dioxygenase gene family
Source: BMC Plant Biol. 2013 Oct 9;13:156. doi: 10.1186/1471-2229-13-156 (PMC3854447; doi:10.1186/1471-2229-13-156)
Supplement: Additional file 2 — Constructs and plasmids used in this study. [file 1471-2229-13-156-S2.pdf]

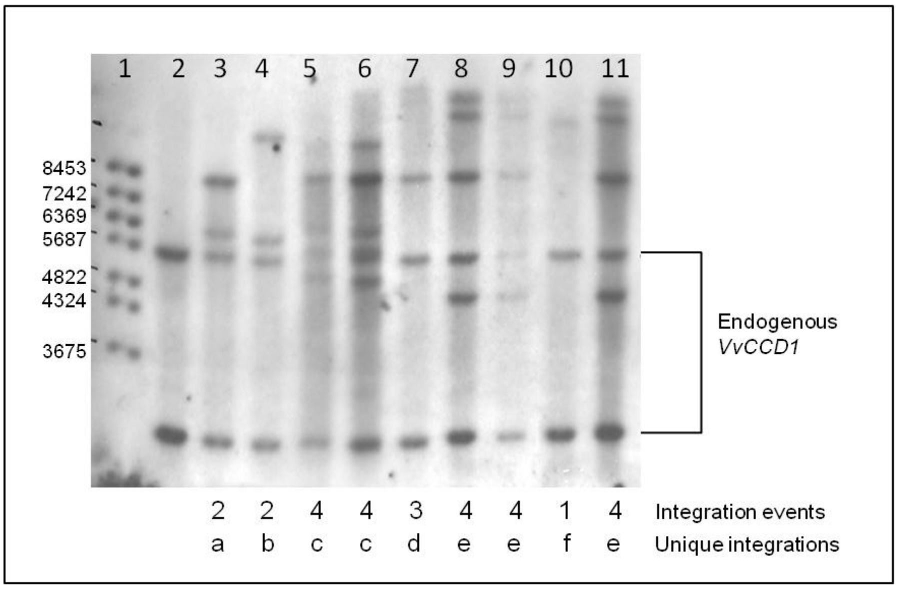

Additional file 3. Southern hybridisation confirming integration of pART27-VvCCD1. Band sizes (in bp) of λ DNA digested with *Bst*E II (lane 1) is shown. Genomic DNA was digested with *Spe*I. Two hybridisation events in the wild-type (lane 2) indicate two copies of *VvCCD1* in the Sultana genome. Lane 3, CCD1-01; lane 4, CCD1-02; lane 5, CCD1-10; lane 6, CCD1-12; lane 7, CCD1-14; lane 8, CCD1-15; lane 9, CCD1-17; lane 10, CCD1-18; lane 11, CCD1-19. Estimated number of integration events are displayed at the bottom of each lane. Plants with the same clonal group (a-f) are considered clonal copies.
