# Supplementary material for: Functional characterisation of three members of the Vitis vinifera L. carotenoid cleavage dioxygenase gene family
Source: BMC Plant Biol. 2013 Oct 9;13:156. doi: 10.1186/1471-2229-13-156 (PMC3854447; doi:10.1186/1471-2229-13-156)
Supplement: Additional file 3 — Southern hybridisation confirming integration of pART27-VvCCD1. Band sizes (in bp) of λ DNA digested with BstE II (lane 1) is shown. Genomic DNA was digested with SpeI. Two hybridisation events in the wild-type (lane 2) indicate two copies of VvCCD1 in the Sultana genome. Lane 3, CCD1-01; lane 4, CCD1-02; lane 5, CCD1-10; lane 6, CCD1-12; lane 7, CCD1-14; lane 8, CCD1-15; lane 9, CCD1-17; lane 10, CCD1-18; lane 11, CCD1-19. Estimated number of integration events are displayed at the bottom of each lane. Plants with the same clonal group (a-f) are considered clonal copies. [file 1471-2229-13-156-S3.pdf]

Additional file 4. Protein characterisation of the carotenoid cleavage dioxygenases from *A. thaliana* and the *V. vinifera* orthologues present in the grapevine genome

**CCD**	**Accession[1]**	**Length (aa)**	**Subcellular localisation[2]**	**Transit peptide[3]**	**pI[4]**	**MW[5]**	**Conserved histidines (aa position)[6]**
AtCCD1	At3g63520	538	Cytosol	-	6.05	60908.11	H_{221}—H_{335}—H_{486}—H_{522}
VvCCD1.1	VV13G12460	542	Cytosol	-	6.14	61364.44	H_{222}—H_{336}—H_{490}—H_{526}
VvCCD1.2	VV13G12530	542	Cytosol	-	5.97	61150.21	H_{222}—H_{336}—H_{490}—H_{526}
AtCCD4	At4g19170	595	Chloroplast; membrane bound	1-30	6.42	65601.73	H_{286}—H_{403}—H_{546}—H_{582}
VvCCD4a	VV02G12270	573	Chloroplast; membrane bound	1-32	6.49	63381.46	H_{269}—H_{383}—H_{524}—H_{560}
VvCCD4b	VV02G12290	589	Chloroplast; membrane bound	1-6	6.63	65607.28	H_{281}—H_{396}—H_{540}—H_{576}
VvCCD4c	VV16G01030	586	Chloroplast; membrane bound	1-8	5.78	65088.35	H_{286}—H_{401}—H_{536}—H_{572}
AtCCD7	At2g44990	629	Chloroplast; membrane bound	1-25	6.30	70850.30	-
VvCCD7	VV15G01930	626	Chloroplast; membrane bound	1-29	7.68	70328.58	-
AtCCD8	At4g32810	570	Chloroplast	1-29	6.65	63956.91	-
VvCCD8	VV04G03390	563	Chloroplast	1-27	7.27	62343.90	-

[1] Accession number for the CCD protein sequences in TAIR (At-) (http://www.arabidopsis.org/) and PLAZA version 1 (At- and Vv-) (http://bioinformatics.psb.ugent.be/plaza_v1/)

[2] Softberry Protcomp (http://linux1.softberry.com/berry.phtml) prediction of the subcellular localisation of the predicted CCD protein sequence

[3] Length (in amno acids) of predicted transit peptide from Softberry Protcomp prediction

[4] Theoretical isolelectric point (pI) of the predicted CCD protein sequence (http://web.expasy.org/compute_pi/)

[5] Theoretical molecular weight (MW) of the predicted CCD protein sequence (http://web.expasy.org/compute_pi/)

[6] Position of the conserved histidines in the predicted CCD protein sequence (from the multiple protein alignments in Supplementary figure X)
