# Supplementary material for: Functional characterisation of three members of the Vitis vinifera L. carotenoid cleavage dioxygenase gene family
Source: BMC Plant Biol. 2013 Oct 9;13:156. doi: 10.1186/1471-2229-13-156 (PMC3854447; doi:10.1186/1471-2229-13-156)
Supplement: Additional file 4 — Protein characterisation of the carotenoid cleavage dioxygenases from A. thaliana and the V. vinifera orthologues present in the grapevine genome. [file 1471-2229-13-156-S4.pdf]

```
VVCCD4a         MDAFSSS-FLSSTFTFPSLTTRP---PIAPSSLPQIPSLNISAVRIEEKQPQSLTAETSS 56
VVCCD4b         MNPLFCP-FLSSTLPHPKPLVSPSLTTTRPSSSPYPPFLHISAIRNVEDKLHSTFYATPT 59
AtNCED4         MDSVSSSSFLSSTFSLHHSLLRR-----RSSS---PTLLRINSAVVEERSPITNPSDNND 52
VVCCD4c         ---MITP-MVSFAFFQIQPLQRS----NQPNSPISFFNGNIAQYCSLPKKNMIIASELST 52
VvCCD1.1        ------------------------------------------------------------
VVCCD1.2        ------------------------------------------------------------
AtCCD1          ------------------------------------------------------------

VVCCD4a         QSSKTQVHKPPPPPPRRAALPTRNIPKKGAAEPSLPVTIFNALDDVINNFIDPPLRSSVD 116
VVCCD4b         TS-------------QFPEIPTTVITAKKRPVPSLLVTIFNGLDDFINNFIGPPLPPSID 106
AtNCED4         RRNK------PKTLHNRTNHTLVSSPPKLRPEMTLATALFTTVEDVINTFIDPPSRPSVD 106
VVCCD4c         SERAP----SPPKLPNIGSDRDTNSEGLGKGALEFLESFLSFFSTSLLYFINPPLHPSVD 108
VvCCD1.1        ----------------MAEKEEQGGTGVVVVDPKPSKGFTSKAVDWLEKLIVKLMYDSSQ 44
VVCCD1.2        ----------------MAEKEEQGGAGVVVVDPTPSKGFTSKAVDWLEKLIVKLMHDSSQ 44
AtCCD1          ----------------MAEKLSDGSS-IISVHPRPSKGFSSKLLDLLERLVVKLMHDASL 43
                                                      : .     :  ::      :

VVCCD4a         PRYVLSQNFAP-VEELPPTECEVTDGSLPPWLDGAYIRNGPNPQFLPRGPYHLFDGDGML 175
VVCCD4b         PKHVLSGNFAP-VDELPPTECEVIEGSLPPCLDGAYIRNGPNPQFYPRGPHHLFDGDGML 165
AtNCED4         PKHVLSDNFAPVLDELPPTDCEIIHGTLPLSLNGAYIRNGPNPQFLPRGPYHLFDGDGML 166
VVCCD4c         PKHVLTGNFAQ-VDELPPIDCLVVEGELPQSLNGTYIRNGPNPLHQPRGPHHLFEGDGML 167
VvCCD1.1        PLHYLSGNFAPVRDETPPCKNLPVIGYLPECLNGEFVRVGPNPKFSPVAGYHWFDGDGMI 104
VVCCD1.2        PLHYLSGNFAPVRDETPPCKNLPVIGYLPECLNGEFVRVGPNPKFSPVAGYHWFDGDGMI 104
AtCCD1          PLHYLSGNFAPIRDETPPVKDLPVHGFLPECLNGEFVRVGPNPKFDAVAGYHWFDGDGMI 103
                * : *: ***   :* ** .     * **  *:* ::* ****  . .  . :* *:****:

VVCCD4a         HSIRISQGRAILCSRYVKTYKYTIERRAGSPILPN------------------------- 210
VVCCD4b         HSIRISHGRPIFCSRYVKTYKYIIEKRAGSPVIPNLFSSYR----SFARSAVAIARLLTG 221
AtNCED4         HAIKIHNGKATLCSRYVKTYKYNVEKQTGAPVMPNVFSGFNGVTASVARGALTAARVLTG 226
VVCCD4c         HSIRLSDGRATFCSRYVKTYKYALEDNVGFPIFPNILSGFHS-VVDLGRCAIAIGRVMKG 226
VvCCD1.1        HGLRIKDGKATYVSRYVRTSRLKQEEYFGGAKFTRFGDLKG--LFGLLMVNMQMLRAKLK 162
VVCCD1.2        HGLHIKDGKATYVSRYVRTSRLKQEEYFGGAKFMRIGDLKG--LFGLLMVNMQMLRAKLK 162
AtCCD1          HGVRIKDGKATYVSRYVKTSRLKQEEFFGAAKFMKIGDLKG--FFGLLMVNVQQLRTKLK 161
                *.::: .*:.   ****:* :   *   * . : .

VVCCD4a         -FNPVNGIGLANTSLALFGGRLYALGESDLPYSLRLKPDGDIETLGRHDFDGKLVMSMTA 269
VVCCD4b         QFDPVNGVGLANTSVAFFCGHLYALAESDLPYAVRLTPDGDIKTLGRYDFDGKLSMSMTA 281
AtNCED4         QYNPVNGIGLANTSLAFFSNRLFALGESDLPYAVRLTESGDIETIGRYDFDGKLAMSMTA 286
VVCCD4c         QIDLRKGFGLANTSLSLFSNRLFALGESDLPYSIHLSEEGDIETIGRCDFDGKAFINMTA 286
VvCCD1.1        ILDVSYGTGTGNTALVYHHGKLLALSEVDKPYVLKVLEDGDLQTLGLLDYDKRLTHSFTA 222
VVCCD1.2        ILDVSYGTGTGNTALVYHHGKLLALSEADKPYVLKVLEDGDLQTLGMLDYDKRLTHSFTA 222
AtCCD1          ILDNTYGNGTANTALVYHHGKLLALQEADKPYVIKVLEDGDLQTLGIIDYDKRLTHSFTA 221
                  :   * * .**::  . .:* ** * * ** :::  .**::*:*  *:* :   .:**

VVCCD4a         HPKVDPETGEAFAFRYGPVPPFLTYFRFDAQGRKQPDVPIFSLTSPSFLHDFGITKKYAI 329
VVCCD4b         HPKIDPSTGEAFAFRYSPVRPFLTYFRFDAQGKKQPDVPIFSLSCPSFFHDFAITNRYAI 341
AtNCED4         HPKTDPITGETFAFRYGPVPPFLTYFRFDSAGKKQRDVPIFSMTSPSFLHDFAITKRHAI 346
VVCCD4c         HPKIDPETGETFAFRCSPIPPYITFFSIDKEGSKQQDVPIFSMTDPTFVHDFSITKQYIV 346
VvCCD1.1        HPKVDPFTGEMFTFGYSHTPPYITYRVISKDGFMHEPVPIT-ISDPIMMHDFAITENYAI 281
VVCCD1.2        HPKVDPFTGEMFSFGYSHTPPYITYRVISKDGFMHEPVPIT-ISDPIMMHDFAITENYAI 281
AtCCD1          HPKVDPVTGEMFTFGYSHTPPYLTYRVISKDGIMHDPVPIT-ISEPIMMHDFAITETYAI 280
                *** ** *** *:*   .   *::*:  :.  *  :  ***  :: * :.***.**: : :

VVCCD4a         FADIQIGM--NPVEMVT-GGSPVGTVPNKVPRLGIIPRYAKDESEMRWFNVPGFNIVHSI 386
VVCCD4b         FPDIQMWM--NPVKMIIRGGSPVGTDPTKVPRVGIIPRYAKDESEMRWIDVPGFNIIHAI 399
AtNCED4         FAEIQLGMRMNMLDLVLEGGSPVGTDNGKTPRLGVIPKYAGDESEMKWFEVPGFNIIHAI 406
VVCCD4c         FSESQIEM--NPLRLMMCKGMPVSAELDKVPRIGVLPRYASTDSEIRWFEAPGFNAMHAI 404
VvCCD1.1        FMDLPLYFR--PKEMVKEKKLIFTFDATKKARFGVLPRYAKNELHIKWFELPNCFIFHNA 339
VVCCD1.2        FMDLPLYFR--PKEMVKEKKLIFTFDATKKARFGVLPRYAKNELHIKWFELPNCFIFHNA 339
AtCCD1          FMDLPMHFR--PKEMVKEKKMIYSFDPTKKARFGVLPRYAKDELMIRWFELPNCFIFHNA 338
                * :  : :      ::           * .*.*::*:**  :  ::*:: *.    .*

VVCCD4a         NAWDEED--AIIMVAPNILSVEHT----LERLDMIHASVEMVRIDLKTGMVTRHPLSTRN 440
VVCCD4b         NAWDEEDGDAIVMVAPNILPIEHA----LERMDLVHGSLEKVRIDLKTGTVTRHRLSQWN 455
AtNCED4         NAWDEDDGNSVVLIAPNIMSIEHT----LERMDLVHALVEKVKIDLVTGIVRRHPISARN 462
VVCCD4c         NAWEEGD-EEIILVAPNAISIENL----FHSIEKVHFSLEKVRINLRSGSVTRTTLSQKN 459
VvCCD1.1        NAWEEEDEVVLITCRLENPDLDLVGRNVKEKLENFANELYEMRFNMKTGIASQRKLSASS 399
VVCCD1.2        NAWEEEDEVVLITCRLENPDLDLVGGDVKEKLENFGNELYEMRFNMKTGIASQRKLSASS 399
AtCCD1          NAWEEEDEVVLITCRLENPDLDMVSGKVKEKLENFGNELYEMRFNMKTGSASQKKLSASA 398
                ***:* *   ::    :    ::        . :: .    :   :::: :* . :   :*

VVCCD4a         LDFAVINPGYVGKKNKYVYAAVGNPMPKISGVVKLDVSQTERK-ECIVGSR-------MY 492
VVCCD4b         LEFAVINPGYLGKKNRYVYSAVGDPLPKISGIVKLDVSRSDRRQECIVAKR-------MY 508
AtNCED4         LDFAVINPAFLGRCSRYVYAAIGDPMPKISGVVKLDVSKGDRD-DCTVARR-------MY 514
VVCCD4c         LELGSINPSYVGKRNRYGYMGIGKMIPKMSGVVKIDL-----ELECEVSRR-------LY 507
```

```
VvCCD1.1        VDFPRVNESYTGRKQRYVYGTILDSIAILDSIAKFDLHAEPDTGKSKLEVGGNVQGIFDL 459
VVCCD1.2        VDFPRVNESYTGRKQRYVYGTILDSIAKVTGIIKFDLHAEPDTGKSKLEVGGNVQGIFDL 459
AtCCD1          VDFPRINECYTGKKQRYVYGTILDSIAKVTGIIKFDLHAEAETGKRMLEVGGNIKGIYDL 458
                :::  :*  : *: .:* *  : . :. : .: *:*:       .  :

VVCCD4a         GPGCYGGEPFFVAREPDNPEAEEDDGYIVSYVHDEKSGESKFLVMDAKTPNLDIVAAVRL 552
VVCCD4b         EPGCYGGEPFFVAKEPDNPEAEEDDGYVLSYVHDEQSGKSRFIVMDAQSPDLDIVAAVKL 568
AtNCED4         GSGCYGGEPFFVARDPGNPEAEEDDGYVVTYVHDEVTGESKFLVMDAKSPELEIVAAVRL 574
VVCCD4c         GAGCFGGEPLFVAKDG---ASEEDDGYIVSYVHDEKSGASRFVVMDAKSQTLDVVAAVKL 564
VvCCD1.1        GVGRFGSEAVFVPREPGI-TSEEDDGYLIFFVHDEKTGKSYVNVINAKTMSPDPVAIVEL 518
VVCCD1.2        GVGRFGSEAVFVPREPGI-TSEEDDGYLIFFIHDEKTGKSYVNVIDAKTMSPDPIAIVEL 518
AtCCD1          GEGRYGSEAIYVPRE----TAEEDDGYLIFFVHDENTGKSCVTVIDAKTMSAEPVAVVEL 514
                  * :*.*..:*.::     :******:: ::*** :* * . *::*::   : :* *.*

VVCCD4a         PRRVPYGFHGLFVRERDIKGL--- 573
VVCCD4b         PTRVPYGFHGLFVKGCDLKMD--- 589
AtNCED4         PRRVPYGFHGLFVKESDLNKL--- 595
VVCCD4c         PRRVPYGFHGLFVKDGDIREIH-- 586
VvCCD1.1        PNRVPYGFHAFFVTEEQLKEQAKL 542
VVCCD1.2        PNRVPYGFHAFFVTEEQLKEQAKL 542
AtCCD1          PHRVPYGFHALFVTEEQLQEQTLI 538
                * *******.:**   ::.
```

Additional file 5. Clustal multiple protein alignments of carotenoid cleavage dioxygenase encoding sequences of *A. thaliana* and (At-) and *V. vinifera* (Vv) orthologues. Arrows indicate the conserved histidine residues.
