# Supplementary material for: Functional characterisation of three members of the Vitis vinifera L. carotenoid cleavage dioxygenase gene family
Source: BMC Plant Biol. 2013 Oct 9;13:156. doi: 10.1186/1471-2229-13-156 (PMC3854447; doi:10.1186/1471-2229-13-156)
Supplement: Additional file 5 — Clustal multiple protein alignments of carotenoid cleavage dioxygenase encoding sequences of A. thaliana and (At-) and V. vinifera (Vv) orthologues. Arrows indicate the conserved histidine residues. [file 1471-2229-13-156-S5.pdf]

Additional file 6. **Functionality and substrate specificity of VvCCD1, VvCCD4a and VvCCD4b in a heterologous *in vivo* bacterial system**. CCDs were expressed in *Escherichia coli* engineered to accumulate specific carotenoids. Carotenoids produced before cleavage were determined using UPLC. Volatile apocarotenoids produced after cleavage were determined using GC-MS.

**Carotenoid accumulating plasmid (pAC-)**	**Carotenoid → Apocarotenoid**	**Cleavage position**	**pTWIN1 (vector control)**	**VvCCD1**	**VvCCD4a**	**VvCCD4b**
			**Average ng.L^{-1} apocarotenoid produced ± standard deviation (n=3)**			
pAC-ZETA	ζ-carotene (80%)[1] → Geranylacetone	9,10(9',10')	803.61±75.20	ND[2]	ND	2255.04±74.57
pAC-NEUR	Neurosporene (100%)[1] → Geranylacetone	(9',10')	388.61±12.73	184.18±53.08	671.43±32.15	646.39±37.01
pAC-LYC	Lycopene (100%)[1] → 6-MHO	5,6(5',6')	393.19±49.42	660.70±44.36	756.00±59.27	782.00±15.45
pAC-EPSILON	ε-carotene (70%)[1] → α-ionone	9,10(9',10')	40.24±8.35	336.15±16.32	190.91±3.57	262.08±1.88
pAC-BETA	β –carotene (100%)[1] → β-ionone	9,10(9',10')	76.28±8.47	349.61±20.46	< LOQ[3]	< LOQ

[1] The percentage of the specific carotenoid substrate present in the strain before VvCCD induction as determined by UPLC analysis

[2] "ND" Not detected

[3] "< LOQ" Below level of quantification
