# Supplementary material for: Functional characterisation of three members of the Vitis vinifera L. carotenoid cleavage dioxygenase gene family
Source: BMC Plant Biol. 2013 Oct 9;13:156. doi: 10.1186/1471-2229-13-156 (PMC3854447; doi:10.1186/1471-2229-13-156)
Supplement: Additional file 6 — Functionality and substrate specificity of VvCCD1, VvCCD4a and VvCCD4b in a heterologous in vivo bacterial system. CCDs were expressed in Escherichia coli engineered to accumulate specific carotenoids. Carotenoids produced before cleavage were determined using UPLC. Volatile apocarotenoids produced after cleavage were determined using GC-MS. [file 1471-2229-13-156-S6.pdf]

Additional file 7. Carotenoids and chlorophyll concentrations present in the grapevine organs investigated in this study. Carotenoids and chlorophylls were analysed by HPLC. Individual carotenoids and chlorophylls were identified by comparison to authentic standards and quantified by normalisation to an internal standard (β-apo-carotenal) and quantified by external standard curve as described in Lashbrooke et al. (2010).

	CAROTENOID AND CHLOROPHYLL CONCENTRATIONS ($ng.g^{-1}$ FW) IN GRAPEVINE ORGANS									
	LEAF		FLOWER		BERRY DEVELOPMENTAL STAGES					
					Green		Véraison		Ripe	
**CHLOROPHYLLS**	**Average**	**± SD (n=3)**	**Average**	**± SD (n=3)**	**Average**	**± SD (n=3)**	**Average**	**± SD (n=3)**	**Average**	**± SD (n=3)**
Chlorophyll a	868480.5	34780.5	288955.6	7169.3	69748.7	2735.3	18402.6	291.5	16380.2	900.5
Chlorophyll b	289905.3	14114.9	119338.4	3130.3	32254.1	1267.4	10615.1	445.3	9907.3	737.1
**Total Chlorophylls**	**1158385.8**	**48895.3**	**408294.0**	**10299.7**	**102002.9**	**4002.6**	**29017.7**	**736.8**	**26287.6**	**1637.6**
**CAROTENOIDS**										
Lutein	97120.7	5702.0	37838.4	931.3	8058.8	399.8	1965.1	79.2	1889.8	95.8
Lutein 5,6 epoxide	0.0	0.0	1627.1	27.7	1331.8	82.1	595.9	14.1	568.3	11.6
β-carotene	62687.3	1358.0	15401.1	792.0	4228.5	111.6	1015.0	58.6	910.8	34.5
Zeaxanthin	97120.7	4701.7	8855.7	197.5	8058.8	0.0	1965.1	231.8	1889.8	298.5
Antheraxanthin	55470.2	4299.9	8396.3	119.3	739.0	53.4	598.5	181.5	540.6	58.7
Violaxanthin	67682.9	4320.8	9355.7	227.6	2857.8	163.7	570.2	60.3	460.9	34.3
Neoxanthin	36191.9	1162.7	13116.1	357.3	3454.5	285.3	828.2	13.2	842.1	57.4
Unknown xanthophyll 1	4957.8	1049.3	983.6	503.3	296.7	40.6	58.2	100.8	193.4	25.5
Unknown xanthophyll 2	0.0	0.0	0.0	0.0	0.0	0.0	37.9	65.6	121.8	85.1
**Total Carotenoids**	**421231.6**	**22594.4**	**95574.0**	**3156.0**	**29025.9**	**1136.5**	**7634.1**	**805.2**	**7417.6**	**701.4**
**Total Xanthophylls**	**353586.5**	**20187.1**	**79189.3**	**1860.6**	**24500.7**	**984.4**	**6523.0**	**580.1**	**6191.6**	**556.2**
