# Supplementary material for: Functional characterisation of three members of the Vitis vinifera L. carotenoid cleavage dioxygenase gene family
Source: BMC Plant Biol. 2013 Oct 9;13:156. doi: 10.1186/1471-2229-13-156 (PMC3854447; doi:10.1186/1471-2229-13-156)
Supplement: Additional file 7 — Carotenoids and chlorophyll concentrations present in the grapevine organs investigated in this study. Carotenoids and chlorophylls were analysed by HPLC. Individual carotenoids and chlorophylls were identified by comparison to authentic standards and quantified by normalisation to an internal standard (β-apo-carotenal) and quantified by external standard curve as described in Lashbrooke et al. (2010). [file 1471-2229-13-156-S7.pdf]

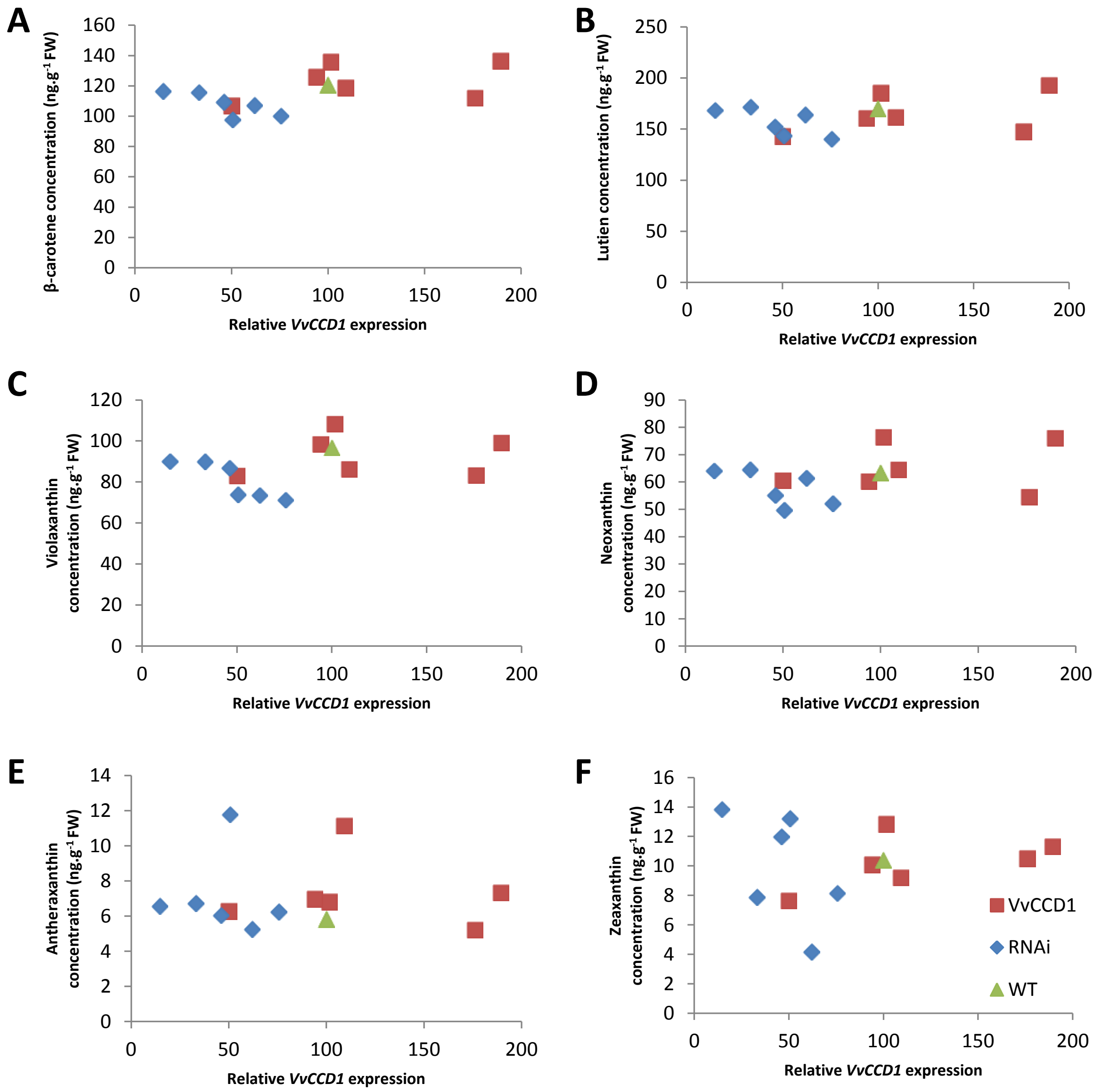

Additional file 8. Relationship between carotenoid concentration and *VvCCD1* expression levels in grapevine leaves. None of the major carotenoids in the leaves of the grapevine population (measured via HPLC) showed significant correlation with *VvCCD1* expression. The concentration of β-carotene (A), lutein (B), violaxanthin (C), neoxanthin (D), antheraxanthin (E) and zeaxanthin (F) found in wild-type (▲), silenced (◆) and overexpression (■) lines is shown.
