# Supplementary material for: Functional characterisation of three members of the Vitis vinifera L. carotenoid cleavage dioxygenase gene family
Source: BMC Plant Biol. 2013 Oct 9;13:156. doi: 10.1186/1471-2229-13-156 (PMC3854447; doi:10.1186/1471-2229-13-156)
Supplement: Additional file 8 — Relationship between carotenoid concentration and VvCCD1 expression levels in grapevine leaves. None of the major carotenoids in the leaves of the grapevine population (measured via HPLC) showed significant correlation with VvCCD1 expression. The concentration of β-carotene (A), lutein (B), violaxanthin (C), neoxanthin (D), antheraxanthin (E) and zeaxanthin (F) found in wild-type (green triangle symbol), silenced (blue diamond symbol) and overexpression (red square symbol) lines is shown. [file 1471-2229-13-156-S8.pdf]

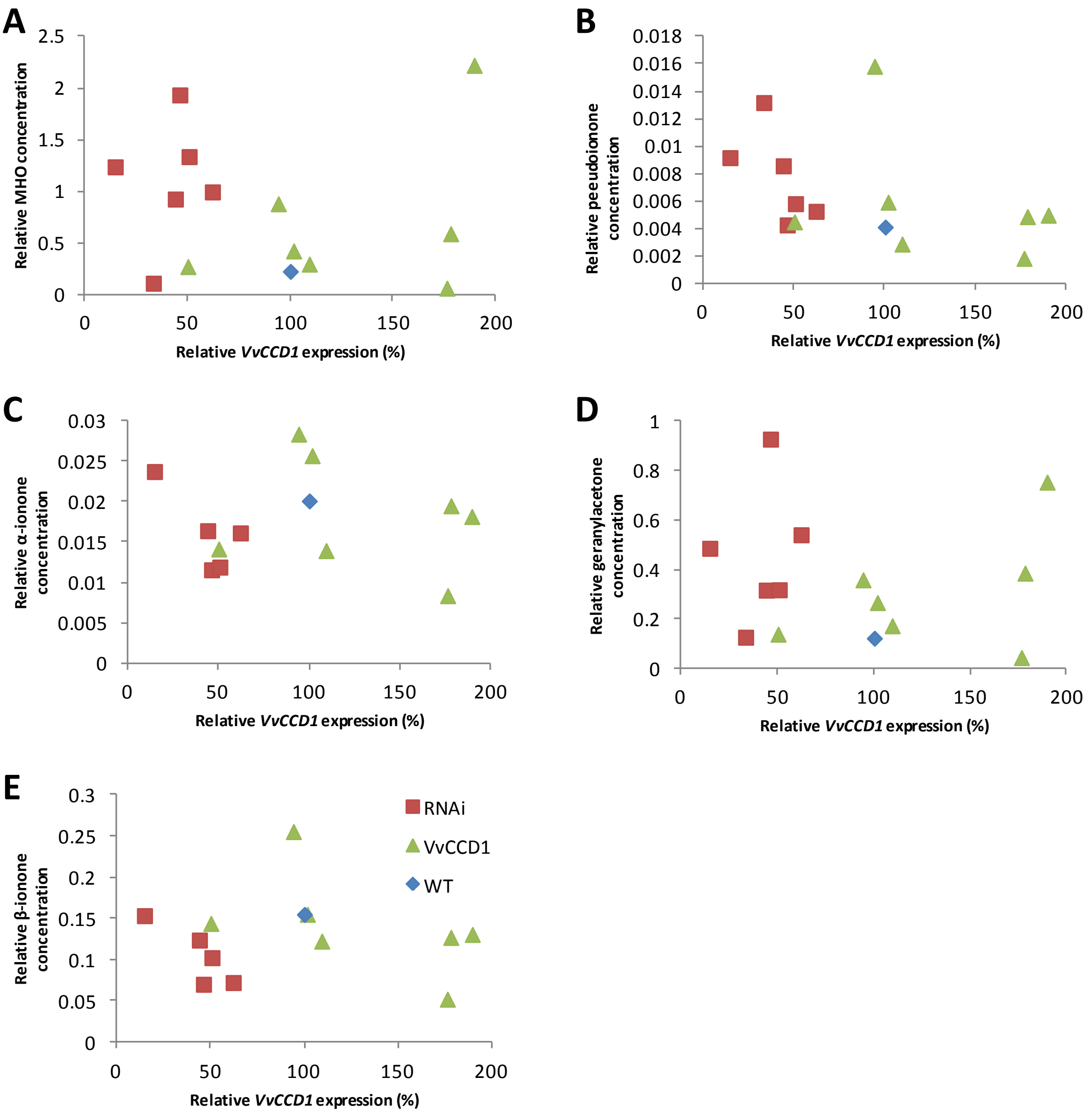


**Additional file 9. Relationship between apocarotenoid concentration and *VvCCD1* expression levels in grapevine leaves.** No significant correlation was found between the *VvCCD1* expression and volatile apocarotenoids in the leaves of the grapevine population (measured via GC/MS). The concentration of 6-methyl-5-hepten-2-one (MHO) (A), pseudoionone (B), α-ionone (C), β-ionone (D), and geranylacetone (E) found in wild-type (◆), silenced (■) and overexpression (▲) lines is shown.
